# Quality of life is substantially worse for community-dwelling older people living with frailty: systematic review and meta-analysis

**DOI:** 10.1007/s11136-019-02149-1

**Published:** 2019-03-14

**Authors:** Thomas F. Crocker, Lesley Brown, Andrew Clegg, Katherine Farley, Matthew Franklin, Samantha Simpkins, John Young

**Affiliations:** 1 Academic Unit of Elderly Care and Rehabilitation, Bradford Institute for Health Research, Bradford Teaching Hospitals NHS Foundation Trust, Temple Bank House, Bradford Royal Infirmary, Duckworth Lane, Bradford, BD9 6RJ UK; 2 Academic Unit of Elderly Care and Rehabilitation, University of Leeds, Bradford, BD9 6RJ UK; 3 Leeds Institute of Health Sciences, University of Leeds, Leeds, LS2 9NL UK; 4 School of Health and Related Research (ScHARR), University of Sheffield, Sheffield, S1 4DT UK; 5 Royal Melbourne Hospital, 300 Grattan St, Parkville, VIC 3050 Australia

**Keywords:** Quality of life, Frailty, Systematic review, Health-related quality of life, Psychological well-being, Subjective well-being

## Abstract

**Purpose:**

Frailty is an important predictor of adverse health events in older people, and improving quality of life (QOL) is increasingly recognised as a focus for services in this population. This systematic review synthesised evidence of the relationship between frailty and QOL in community-dwelling older people, with an emphasis on how this relationship varied across QOL domains.

**Methods:**

We conducted a systematic review with meta-analysis. We searched five databases for reports of QOL in older people with frailty and included studies based on pre-defined criteria. We conducted meta-analyses comparing “frail” and “not frail” groups for each QOL scale where data were available. We compared pooled results to distribution-based and known-group differences to enhance interpretation. We summarised reported cross-sectional and longitudinal analyses.

**Results:**

Twenty-two studies (24,419 participants) were included. There were medium or larger standardised mean differences for 24 of 31 QOL scales between frail and not frail groups, with worse QOL for frail groups. These scales encompassed constructs of health-related quality of life as well as psychological and subjective well-being. There were similar findings from mean difference meta-analyses and within-study analyses.

**Conclusions:**

The association between frailty and lower QOL across a range of constructs is clear and often substantial. Future research should establish whether causal mechanisms link the constructs, which aspects of QOL are most important to older people with frailty, and investigate their tractability. Services focused on measuring and improving QOL for older people with frailty should be introduced.

**Electronic supplementary material:**

The online version of this article (10.1007/s11136-019-02149-1) contains supplementary material, which is available to authorized users.

## Introduction

Enhancing Quality of Life (QOL) has been an explicit or implicit goal for individuals, communities, nations and the world [1]. QOL is a complex concept and its precise formulation is contested [1–3]. It is defined by the World Health Organization (WHO) as: “An individual’s perceptions of their position in life, in the context of the culture and value systems in which they live, and in relation to their goals, expectations, standards and concerns” [4]. The global demographic transition to older populations has meant health care organisations internationally have adopted a greater focus on enhancing QOL for older people [5]. Indeed, prioritising QOL in later life, in preference to disease-based outcomes, is consistent with the views of older people themselves [6, 7]. QOL measures can help estimate the needs of a population and improve clinical decision making, resource allocation and policy [8–10], and QOL assessments are increasingly collected in studies involving older people [11–13].

The concept of frailty as an abnormal health state characterised by loss of biological reserves related to the aging process has emerged in the last 15 years. It has proved a better discriminator than chronological age in the prediction of mortality and variations in outcomes in later life [14], and robust models have been developed and validated to identify frailty [15]. Approximately, one in ten people over 65 years, and between a quarter and a half of those aged over 85 years, are living with frailty [15]. There is an argument that frailty should be considered and managed as a long-term condition [16] and assessments for frailty are increasingly being incorporated into routine practice to ensure both its improved detection and the subsequent delivery of care that gives greater emphasis to QOL for older people living with frailty.

The relationship between frailty and QOL has attracted research interest, though the findings have been inconsistent. A systematic review reported an inverse relationship between frailty and QOL among community-dwelling older people [17]. However, there were limitations with the review, notably the limited use of meta-analysis, little consideration of differing constructs of QOL and inclusion of data from intervention studies, which are prone to selection bias, producing unrepresentative samples [18]. We have therefore conducted a further systematic review to investigate the impact of frailty on QOL, and vice versa, but with a particular focus on the domains of QOL that are most affected. We anticipate that this information will facilitate more targeted approaches for interventions for older people with frailty.

## Methods

### Study inclusion criteria

We conducted a systematic review with meta-analysis. We included cohort or cross-sectional studies. Studies where the data were part of an intervention study, even if collected prior to intervention, were excluded. We included studies if the participants were community-dwelling older people (mean age ≥ 65 years). We included studies that reported QOL by frailty status, or association between frailty and QOL.

We included studies with a validated instrument for frailty. Because of the diverse definitions for QOL, we included studies with instruments which were described as measuring “quality of life”, “well-being” or “life satisfaction” by a study that met the other inclusion criteria, or where this was implied by the name of the instrument itself, such as The World Health Organization *Quality of Life* (WHOQOL) scale [4].

We only included studies with reports written in English, or where authors could provide data in a format we could utilise.

### Search strategy

We developed a search strategy with an information specialist using controlled vocabulary and text words to search databases including AMED, CINAHL, MEDLINE and Web of Science (last searched 4 April 2017; see Online Resource 1).

We adopted an iterative search procedure, updating our strategy and repeating our searches to incorporate specific terms for each instrument that at least one study identified as a measure of QOL, to ensure that we identified and pooled all available data.

### Data collection

Two reviewers independently conducted each stage of study selection, data extraction and assessment of risk of bias and compared results. Disagreements were resolved by discussion with other members of the review team. Study authors were contacted for further information where necessary.

### Selection of studies

We assessed the titles and abstracts from the electronic searches against the stated eligibility criteria. We obtained full text articles of potentially eligible studies and assessed these against the criteria to determine study inclusion. This process was repeated each time additional QOL instruments were identified.

### Data extraction and management

We extracted data using a pre-specified and piloted form (see Online Resource 2 for template of electronic form). Where scores for a particular instrument were reported inconsistently, we standardised their scaling (e.g. transforming WHOQOL-BREF scores to 0–100).

### Risk of bias in individual studies

We assessed risk of bias at the study level using the modified Newcastle–Ottawa Scale [19, 20]. Studies were assessed on the domains of selection, comparability, exposure (i.e. frailty) and outcome (i.e. QOL). Outcome assessment scored one star for self-report because of the appropriateness of this for QOL measurement. The maximum achievable score (least risk of bias) was eight stars. Scores of five stars or more were considered moderate to good quality, although studies were incorporated in the synthesis regardless of rating [20].

### Data synthesis

We calculated and pooled standardised mean differences (SMDs) for each QOL scale using inverse-variance random-effects meta-analysis, where feasible. We grouped participants as “frail” versus “not frail” for these purposes; where other groupings were given, we assigned moderate and severe frailty as “frail” and fit, robust, pre-frail, vulnerable and mild frailty as “not frail”. We pooled mean differences (MDs) for each QOL scale where data were reported in multiple studies following the approach above. Meta-analyses were conducted in Review Manager 5.3 [21].

QOL instruments have a variety of score ranges. Even when ranges are consistent, scores on different scales cannot usually be meaningfully compared, including subscales of the same instrument. Additionally, minimal clinically important differences (MCIDs) are not well established. Therefore, to aid interpretation, we have (1) described SMDs using standard rules of thumb for effect sizes (e.g. small, medium, large; detailed in Online Resource 3) [22]; (2) compared pooled MDs with reference values calculated from general populations in large-scale studies where possible (see Online Resource 3).

We summarised cross-sectional and longitudinal analyses reported in included studies of association between QOL and frailty measures with a narrative synthesis.

## Results

### Study selection

**Fig. 1:**
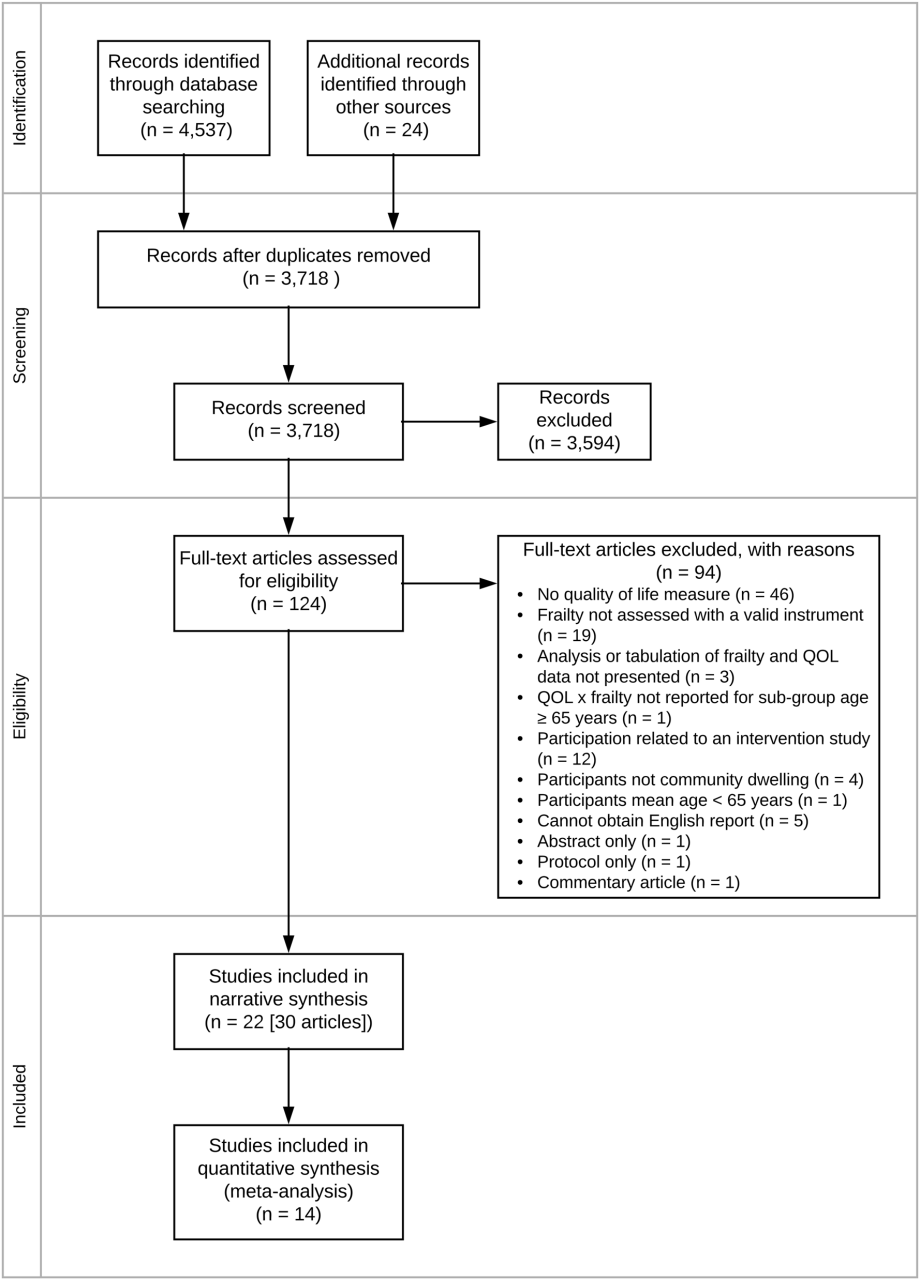
PRISMA flow diagram

See Fig. 1 for the study selection process. We identified 4537 records through database searches and 24 additional references from other systematic reviews. Twenty-one groups of participants were included in the review, reported in 30 articles [23–52]. For one group of participants (the English Longitudinal Study of Aging [ELSA] cohort), we present the results as two separate studies, as differing frailty instruments, age limits and timepoints were used [29, 30]. Therefore, 22 studies were included in the review [23–44].

### Study characteristics

Study characteristics are presented in Table 1.

**Table 1:** Characteristics of included studies

Study name and sources	Aims of study	Study type^a^	Country and years data collected	Sample characterisation	Age mean (SD)	Female %	N^b^	Frailty instrument	QOL instrument/description
Ament 2014 [23]	Study whether physically frail older people are more at risk for developing IADL disability, decreased quality of life, and hospital admission if they also suffer from cognitive, social, or psychological frailty.	→	Netherlands 2010; 2011	older people with frailty	78.1 (4.9)	60%	334	Groningen Frailty Indicator	“Quality of life” (unreferenced single-item general assessment)
Bilotta 2010 [24, 45]	Investigate dimensions and correlates of QOL associated with frailty status among community-dwelling older outpatients.	✕	Italy 2009	Community-dwelling outpatients aged 65 + referred to a geriatric medicine clinic by GP, excluding patients with severe cognitive impairment	81.5 (6.3)	69%	239	Study of Osteoporotic Fractures criteria	Older People’s Quality of Life questionnaire
CSHA [25, 52]	Test the hypothesis of the frailty identity crisis by studying whether psychological well-being was related to frailty and mortality in a sample of older community-dwelling Canadians. Examine the relationship between psychological well-being and depression in older adults and the relative contribution these psychological factors have on risk of functional disability, frailty, and mortality.	→	Canada 1996–1997; 2001–2002	English- and French-speaking Canadians aged 65 years and older. The longitudinal analyses were restricted to community-dwelling older adults without dementia	79.1 (6.4)	61%	5703	Cumulative Deficit Model (33 deficits frailty index)	Ryff Psychological Well-Being scale
Chang 2012 [26]	Identify the incidence of frailty and to investigate the relationship between frailty status and health-related quality of life (HRQoL) in the community-dwelling elderly population who utilise preventive health services.	✕	Taiwan 2011	older people following an extended hospital episode of care	74.6 (6.3)	53%	374	Fried phenotype model	SF-36 “health-related quality of life”
Chang 2016 [27]	Examine the independent effect of frailty on quality of life of community-dwelling older adults	✕	Taiwan years not reported	community-dwelling older people	74.8 (7.0)	57%	239	Study of Osteoporotic Fractures criteria	WHOQOL-BREF
Coelho 2015 [28]	Present the translation and validation process of the Portuguese version of the Tilburg Frailty Indicator	✕	Portugal 2013	Community-dwelling. Users of institutions such as social, recreation and day care centres and senior academies.	79.2 (7.3)	76%	252	Tilburg Frailty Indicator	WHOQOL-OLD; EUROHIS-QOL
ELSA (Gale 2014) [29]	Identify whether psychological well-being was associated with incidence of physical frailty	→	UK 2004–2005; 2008–2009	People aged ≥ 60 years	70.2 (7.7)	55%	2557	Fried phenotype model	CASP-19 “psychological well-being”
ELSA (Hubbard 2014) [30] +	First, to investigate the association between frailty and subjective well-being in older people; second, to explore the impact of household wealth and income on this relationship.	✕	UK 2002–2003	Community-dwelling adults aged 65–79	71	52%	3206	Cumulative Deficit Model (50 deficits frailty index)	CASP-19 “subjective well-being”
Freitag 2016 [31]	Adaptation of the TFI to a German version and to test the reliability and validity of the German adaptation of the TFI in a sample of older adults	✕	Germany 2012	Older adults living at home	75.3 (5.7)	62%	210	Tilburg Frailty Indicator	SF-12 “health-related quality of life”; EUROHIS-QOL
Gobbens 2012 [32, 46, 47]	To assess the predictive validity of frailty and its domains (physical, psychological and social), as measured by the Tilburg Frailty Indicator (TFI), for the adverse outcomes disability, health care utilisation and quality of life	→	Netherlands 2008; 2009; 2010	community-dwelling persons aged 75 years and older	80.3 (3.8)	57%	479; 336; 266	Tilburg Frailty Indicator	WHOQOL-BREF
Gobbens 2013 [33, 48] +	Test the hypothesis that the prediction of quality of life by physical frailty components is improved by adding psychological and social frailty components	✕	Netherlands 2009–2010	Dutch older people who voluntarily complete a web-based questionnaire	73.4 (5.8)	33%	1031	Tilburg Frailty Indicator	WHOQOL-BREF
Jurschik 2012 [34]	Assess the prevalence of frailty and to identify factors associated with frailty in older people living in the community through a cross-sectional study of community-dwelling persons age 75 and older	✕	Spain 2009–2010	community-dwelling persons aged 75 and older	81.3 (5.0)	60%	640	Fried phenotype model	SF-36 “health-related quality of life”
Kanauchi 2008 [35]	Examine the health-related quality of life (HRQOL) and the effect of frailty in elderly patients with cardiometabolic risk factors	✕	Japan 2007	elderly patients with cardiometabolic risk factors (diabetes, hypertension, dyslipidaemia or chronic kidney disease)	72.9 (5.1)	44%	101	Hebrew Rehabilitation Center for Aged Vulnerability Index and the Vulnerable Elders Survey	WHOQOL-BREF
Lahousse 2014 [36] +	Investigate the prevalence of frailty in a Dutch elderly population and to identify adverse health outcomes associated with the frailty phenotype independent of the comorbidities	✕^c^	Netherlands 2009–2012	population-based cohort study in persons aged ≥ 55 years	74 [9] ^d^	56%	2833	Fried phenotype model	EuroQol Visual analogue scale (EQ-VAS) “quality of life”
Lenardt 2014 [37, 49] +	Identifying the quality of life of frail elderly patients, users of primary care services	✕	Brazil 2013	Elderly patients of a primary health care service	70.9 (7.4)	51%	203	Fried phenotype model	SF-36 “quality of life”
Lin 2011 [38, 50] +	Examine the domains and degrees of functioning and well-being that are affected by the frailty of elders residing in the community in Taiwan	✕	Taiwan 2009	Population-based survey of elders residing in the community	73.9	48%	903	Fried phenotype model	SF-36 “health-related quality of life”
Masel 2009 [39, 51]	Elicit the relationship between being non-frail, pre-frail or frail and HRQOL in a representative sample of older Mexican Americans	✕	USA 2005–2006	Representative sample of older Mexicans living in Texas	82.3 (4.5)	64%	1011	Fried phenotype model	SF-36 “health-related quality of life”
Pinto 2016 [40]	Identify the influence of self-rated health as a mediator of the relationships between objective indicators of physical and mental health, as well as the elderly’s life satisfaction	✕	Brazil years not reported	People aged > 65, excluding severe cognitive impairment, low mobility, sequelae of stroke, Parkinson’s disease, severe deficits in hearing, vision, communication or being terminally ill	72.7 (5.4)	66%	2164	Fried phenotype model	“Life satisfaction” (unreferenced multi-item)
Simone 2013 [41]	examine differences in leisure activity engagement by frailty status, and evaluate the link between functional status and subjective well-being	✕	USA years not reported	older people	74 (10.5)	82%	95	Groningen Frailty Indicator	Satisfaction With Life Scale
St John 2013 [42]	determine if (1) frailty is associated with life satisfaction (LS) in community-dwelling older adults in cross-sectional analyses; (2) frailty predicts LS five years later and (3) specific domains of LS are preferentially associated with frailty	→	Canada years not reported	older people	77.5	59%	988	Brief frailty measure and Frailty Index	Life Satisfaction terrible-delightful scale
Wu 2013 [43]	Test and validate a Chinese Taiwan version of the CASP-19 and to analyse its psychometric properties in a community-dwelling older Chinese (Taiwanese) population in Taipei City, Taiwan	✕	Taiwan 2010	sample came from the community-dwelling older participants of a senior citizen’s health examination in Taipei City Hospital, Renai Branch in 2010 or earlier	75.5 (6.5)	50%	699	Chinese Canadian study of health and aging clinical frailty scale	CASP-19 “quality of life”
Yang 2016 [44]	Examine the relationship between frailty and life satisfaction and the roles of age and social vulnerability underlying the links in Chinese older adults	✕	China 2013	Older adults in Shanghai	75.2 (7.6)	54%	1970	Cumulative Deficit Model (52 deficit frailty index)	“Life satisfaction” (unreferenced multi-item)

^a^✕ cross-sectional; → longitudinal

^b^The number of participants in the main analyses of the variables of interest

^c^The article reports longitudinal data but only baseline analyses of the variables of interest

^d^median [IQR]

^+^Additional data provided by authors

### Design

Five studies presented longitudinal analyses incorporating QOL and frailty data [23, 24, 29, 32, 42]. The remaining studies presented only cross-sectional data or analyses for QOL and frailty variables. Nineteen studies examined the relationship between frailty and QOL [23–27, 29, 30, 32–42, 44], two studies assessed the psychometric properties of a frailty instrument [28, 31] and one study assessed the psychometric properties of a QOL instrument [43].

### Setting

Data were collected from populations in 12 countries across Europe, Asia and North and South America. The first wave of data was collected in the 1990s by one study [24], in the 2000s by ten studies [25, 29, 30, 32–36, 38, 39] and in the 2010s by seven studies [23, 26, 28, 31, 37, 43, 44], with four studies not reporting date of data collection [27, 40–42].

### Participants

The included studies report data from 24,419 participants in total (median: 479 participants; smallest study, 95 [41]; largest study, 5703 [24]; not double-counting ELSA cohort participants). The overall mean age (composite standard deviation) was 76.1 (7.5) years where such data were provided. There were 13,905 female participants (57%; study range 33–82%).

Most studies recruited participants from open adverts or mailouts; five studies recruited directly through health services [25, 26, 35, 37, 43].

### Frailty ascertainment

Frailty status within studies was ascertained using: Fried phenotype criteria (eight studies [26, 29, 34, 36–40]; Tilburg Frailty Indicator (four [28, 31–33]); cumulative deficit model (three [24, 30, 44]); Study of Osteoporotic Fractures criteria (two [25, 27]); Groningen Frailty Indicator (two [23, 41]); the Chinese Canadian study of health and aging clinical frailty scale [43]; the Hebrew Rehabilitation Center for Aged Vulnerability Index and the Vulnerable Elders Survey [35] and the Brief frailty measure and Frailty Index [42].

### QOL instruments

Descriptions of the QOL instruments used in the studies are provided in Online Resource 4. Twenty studies reported QOL estimates based on a single instrument only; two studies used more than one instrument [28, 31]. Eight of the instruments have multiple scales (i.e. dimensions).

QOL within studies was measured using: Medical Outcomes Study 36-item Short-Form health survey (SF-36, five studies [26, 34, 37–39]); World Health Organization’s Quality of Life short-form instrument (WHOQOL-BREF; four [27, 32, 33, 35]); Control, Autonomy, Self-realisation and Pleasure, 19-item questionnaire (CASP-19; three [29, 30, 43]); European Health Information Surveys project 8-item QOL index (EUROHIS-QOL; two [28, 31]); 12-item Short-Form survey (SF-12) [31]; Older Adults WHOQOL module (WHOQOL-OLD) [28]; 18-item version of Ryff’s Psychological Well-Being scale [24]; Older People’s Quality of Life Questionnaire (OPQOL) [25]; Satisfaction with Life Scale (SWLS) [41]; EuroQol visual analogue scale (EQ-VAS) [36]; a modified Life-Satisfaction Terrible-Delightful scale [42] and three unreferenced instruments: a single-item evaluation of QOL [23], and two different multi-item life satisfaction instruments [40, 44].

### Risk of bias within studies

The median score of the modified Newcastle–Ottawa Scale was 5 (range 2 to 6). Most studies recruited broad, representative samples, but few provided sample size calculations or described comparability with non-respondents. Most studies provided adjustment for relevant factors and conducted appropriate analyses that were sufficiently reported.

### Synthesis of results

#### Standardised mean difference meta-analyses

**Fig. 2:**
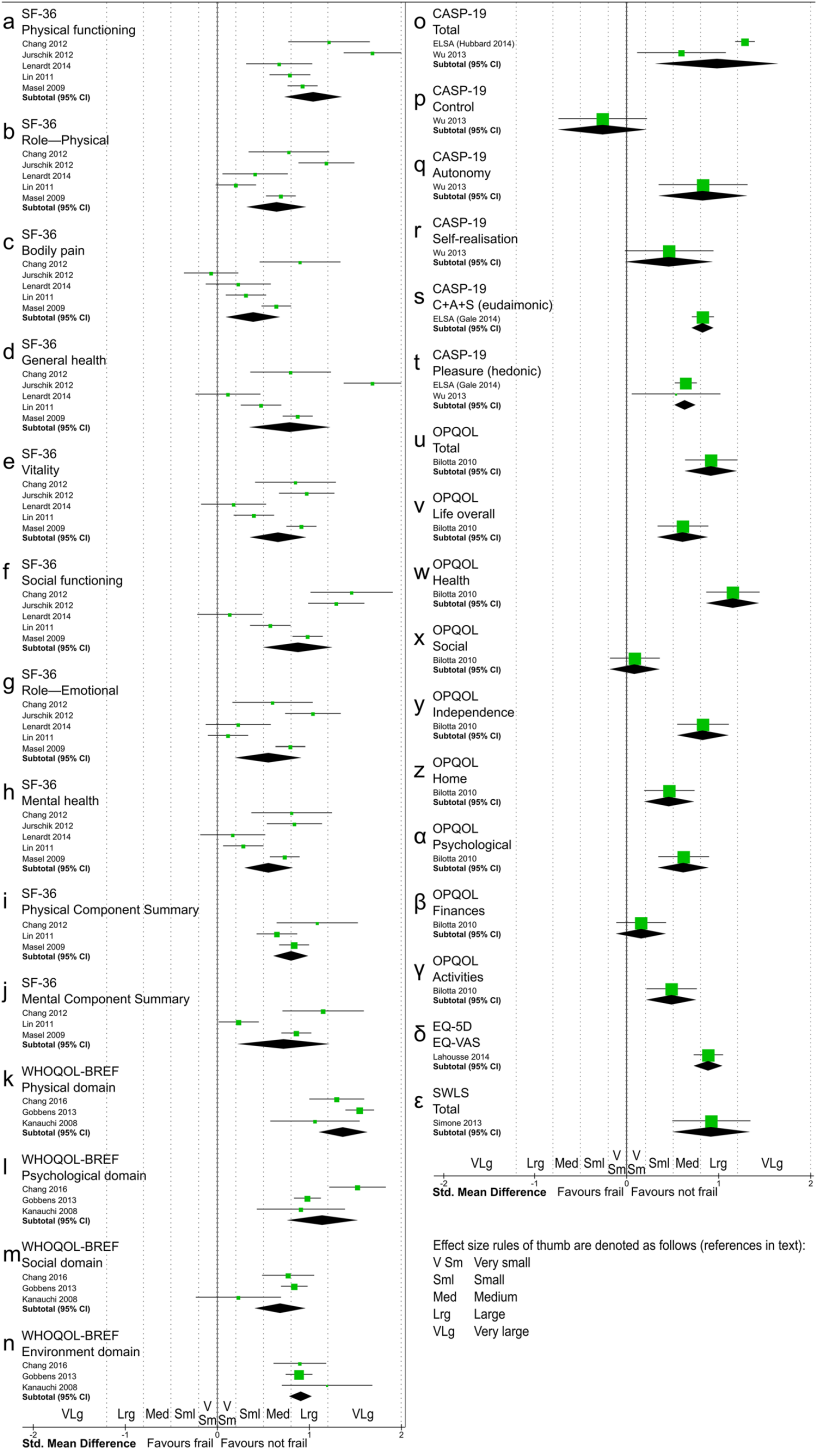
Forest plot of SMD of QOL instruments for frail versus not frail participants using an inverse-variance random-effects model meta-analysis

Random-effects meta-analyses estimated statistically significant SMDs in favour of the ‘not frail’ group for 27 of 31 scales, of which the SMD size were: very large for the WHOQOL physical domain (Fig. 2k), large for 13 scales (SF-36 Physical functioning, SF-36 Social functioning, SF-36 Physical Component Summary, WHOQOL-BREF Psychological, WHOQOL-BREF Environment, CASP-19 Total, CASP-19 Autonomy, CASP-19 C + A + S [eudaimonic], OPQOL Total, OPQOL Health, OPQOL Independence, EQ-VAS, SWLS; Fig. 2a, f, i, l, n, o, q, s, u, w, y, δ and ε), medium for ten scales and small for three scales (forest plots in Fig. 2, additional data in Online Resource 5) [22]. Among the other four scales, SMDs were small for one scale (Fig. 2r), very small for two scales (Fig. 2x and β) and small favouring people with frailty for the CASP-19 control scale (Fig. 2p). Estimates of SMD were insufficiently precise for the upper and lower bounds of 95% confidence intervals to have the same rule of thumb interpretation except for one scale (Fig. 2t, CASP-19 pleasure scale, medium difference).

There was evidence of substantial heterogeneity between studies (*I*^2^ > 20%) for 14 of the 16 scales with more than one study contributing to the meta-analysis. Additionally, there was evidence of substantial heterogeneity between QOL scales (*I*^2^ = 78%). Due to the limited number of studies contributing to each QOL scale and many plausible sources of heterogeneity, we did not investigate heterogeneity through subgroup analyses.

#### Mean difference meta-analyses

Pooled results of studies reporting the SF-36 and WHOQOL-BREF found clinically and statistically significant differences between frail and not frail groups for each QOL scale. Forest plots and data tables for MD meta-analyses are presented in Online Resource 6.

#### Individual study cross-sectional analyses

Measures and analyses from individual studies are reported in Online Resource 7.

Fourteen studies reported on statistical significance of bivariate associations between measures of frailty and QOL. These were statistically significant (*p* < 0.05) for all reported analyses in eleven studies [26–29, 31, 32, 39–43], but only for some QOL scales in the other three studies’ analyses [25, 34, 37].

In 13 studies [24–27, 30, 34–36, 38–40, 42, 44], there were multivariate cross-sectional analyses, with statistically significant associations between frailty and QOL in 44 of the 58 analyses. Those analyses with non-significant results were characterised by small numbers of participants and large numbers of additional variables suggesting they may have been over-adjusted [24, 27, 34, 35].

#### Longitudinal analyses

Four studies reported longitudinal analyses. In Gale 2014, 4-year frailty was associated with baseline CASP-19 total, hedonic and eudaimonic scores in a model adjusted for variables including baseline frailty and depressive symptoms [relative risk of frailty (95% CI) 0.62 (0.52 to 0.74); 0.70 (0.59 to 0.82) and 0.64 (0.53 to 0.76) per SD increase in respective CASP-19 scores] [29]. However, in the Canadian Study of Health and Aging, 5-year frailty was not associated with Ryff’s psychological well-being scale; associations were mediated via depression [52]. Gobbens 2012 reported that addition of baseline frailty to a multivariable model explained an additional 3.7%, 4.4%, 4.6% and 1.8% of the variance in 2-year physical, psychological, social and environmental WHOQOL scores, respectively [32]. Furthermore, Gale 2014 reported that reduced 4-year CASP-19 scores were associated with increased incidence of pre-frailty and frailty among those fit at baseline in adjusted models [29]. According to St John 2013, 5-year life satisfaction domains and life satisfaction overall were explained by baseline frailty status, although unlike other analyses presented here the model was not adjusted for baseline values of the outcome variable nor were the results significant for the life satisfaction domains of housing and self-esteem [42].

### Risk of bias across studies

Visual inspection of funnel plots revealed no evidence of asymmetry, indicating no evidence of publication bias.

## Discussion

To investigate the QOL of community-dwelling older people with frailty, we systematically reviewed the literature and identified 22 observational studies (24,419 participants) that met our broad inclusion criteria. Evidence indicates that people with frailty have worse QOL than people without frailty, with medium to large differences between the groups. This association is robust to adjustment for relevant variables such as age, gender and depression.

QOL is a complex concept and we anticipated diversity in the instruments used in the included studies. Fourteen instruments were used in the 22 studies, many with multiple scales that targeted a wide range of constructs including those focused on health (e.g. limitations in activities, pain and mental health) as well as broader conceptions of well-being such as psychological well-being (e.g. sense of control and self-acceptance); satisfaction with relationships or circumstances (e.g. housing, finances or transport) and overall life-satisfaction and QOL. Our findings therefore relate to QOL as a broadly defined concept and imply that it is a valid outcome for the attention of service providers and researchers in relation to older people with frailty. Future studies should explain their choice of instrument and the importance of the embedded constructs to people with frailty. Ideally, older people with frailty should be involved in instrument selection.

While the point estimate of SMD was medium to large for many QOL scales, there were some notable exceptions and patterns. Physical functioning and satisfaction with health scales were among those with the largest difference (in particular WHOQOL-BREF Physical, SF-36 Physical functioning, OPQOL Health and EQ-VAS), which is perhaps unsurprising given the conceptual overlap with frailty. More broadly, total scores of the OPQOL, CASP-19 and SWLS also had large differences. However, there were inconsistent results for social scales, with the OPQOL Social scale being non-significant and its confidence interval not overlapping those of either the SF-36 Social functioning (large SMD) or WHOQOL-BREF Social (medium SMD) scales. Similarly, there are divergent results for the conceptually similar WHOQOL-BREF Environment scale (large SMD) and the OPQOL Home and Finances scales (small/non-significant SMDs). While there are some differences in focus between the instruments, it would be useful to see if the OPQOL results (currently based on a single study) are repeated, and if so whether it points to aspects with differing importance or perception between people with and without frailty, or perhaps whether it is indicative of problematic scales.

This systematic review updates the earlier review [17] and includes eleven additional studies [23, 27, 28, 34–37, 40–42, 44]. By using an SMD approach, we were able to compare across QOL scales, including a greater number in meta-analyses than the previous review which included only the SF-36 PCS and MCS. The method of calculating the PCS and MCS can lead to anomalous results such as higher MCS in people with lower physical and mental subscales due to the inclusion of all eight SF-36 subscales and the use of negative weights in calculation of each summary score, meaning they should be interpreted in conjunction with the subscales [53, 54]. Our synthesis enabled this, allowing the identification of small to large SMDs across these health-related subscales. We were also able to identify a relatively consistent effect across other QOL constructs. However, our analyses were limited by dichotomising the whole population as ‘frail’ or ‘not frail’, rather than including a pre-frail category, which would have necessitated only examining portions of the population in each analysis or double-counting.

There was a lack of conceptual clarity in some of the studies, as their frailty and QOL definitions overlapped substantially. For example, the Fried criteria of exhaustion, low energy expenditure, slow walking speed and weak grip strength has much in common with the vitality and physical functioning subscales of the SF-36. Similarly, two studies used the Tilburg Frailty Index in conjunction with the WHOQOL-BREF, which contain similar domains. Nevertheless, other studies identified associations across distinct constructs such as between a cumulative deficit model of frailty and the CASP-19.

We were limited in the longitudinal data that were available from the studies, which limited our ability to examine causality. However, there is some evidence that lower psychological well-being may cause incident frailty and that frailty may cause reductions in multiple QOL domains. Evidence of a bi-directional relationship should be treated with caution at this stage. Future research should explore the relationship between QOL overall, factors that contribute to QOL, well-specified models of frailty and proposed mechanisms linking QOL and frailty [e.g. 55–58] using panel data with multiple time points in multilevel models to help disentangle the associations identified in this systematic review. Research such as this would enable better understanding of whether, for example, protecting psychological well-being may lessen frailty, and whether combating frailty could improve QOL. Experimental research is also required to investigate the extent to which QOL is a tractable outcome for this population.

There has been half a century of conceptual thinking and field research about QOL and over two decades relating to frailty. It is now more widely recognised that modern medical interventions applied to older people with frailty can result in unintended harms rather than benefits [15]. Indeed, simply applying standard long-term condition guidelines leads to excessive treatment burdens for older people with multiple chronic conditions [59]; a situation that is commonplace in later life [60]. New care paradigms for older people with frailty are being advanced in Europe [61] and elsewhere [62] in which there is a reframing of service goals and outcomes with a greater emphasis on individualised, person-centred approaches. In the future, health services for older people with frailty will extend the traditional medical approaches to address more of the things that matter to older people [7] and emphasise linking the person to their local community [63].

The findings from our review are therefore reassuring: frailty and QOL are negatively associated with large differences by frailty status for a wide range of QOL constructs. This is important for research funders and service planners who should feel confident to commission, design and introduce novel services with an explicit focus on measuring and improving QOL outcomes for older people with frailty.

## Electronic supplementary material

Below is the link to the electronic supplementary material.

Supplementary material 1 (PDF 179 KB)

Supplementary material 2 (PDF 23 KB)

Supplementary material 3 (PDF 140 KB)

Supplementary material 4 (PDF 208 KB)

Supplementary material 5 (PDF 839 KB)

Supplementary material 6 (PDF 636 KB)

Supplementary material 7 (PDF 208 KB)
