# Supplementary material for: Quality of life is substantially worse for community-dwelling older people living with frailty: systematic review and meta-analysis
Source: Qual Life Res. 2019 Mar 14;28(8):2041–56. doi: 10.1007/s11136-019-02149-1 (PMC6620381; doi:10.1007/s11136-019-02149-1)
Supplement: Supplementary file 1 — Supplementary material 1 (PDF 179 KB) [file 11136_2019_2149_MOESM1_ESM.pdf]

Search strategies by database

AMED (Allied and Complementary Medicine)

Search Strategy:
----
1 "quality of life"/
2 health status/
3 health survey/
4 health outcome*.tw.
5 quality of life.tw.
6 (QOL or "*qol*").tw.
7 (wellbeing or "well being").tw.
8 (HRQL or HRQOL).tw.
9 quality adjusted life year*.tw.
10 (QALY* or "*qaly*").tw.
11 Health state.tw.
12 health status.tw.
13 health* year* equivalent*.tw.
14 life quality.tw.
15 (((short form or shortform) adj3 "36*") or (sf36* or sf-36*)).tw.
16 (sf12* or sf-12* or sf8* or sf-8* or sf6* or sf-6*).tw.
17 (Euroqol or Euro qol or EQ-5D* or EQ5D* or EQ-VAS* or EQVAS*).tw.
18 (Quality of Wellbeing Index or Quality of Well Being or QWB*).tw.
19 (Health utility or Health Utility Index or HUI).tw.
20 (HYE or HYES).tw.
21 (SWB or PWB or GWB).tw.
22 (Medical Outcome* adj (study or survey) adj3 36*).tw.
23 (Rosser adj3 (index* or indicator* or score* or scale* or tool* or test* or model* or phenotype* or criteri* or marker* or method* or instrument* or assess* or exam* or evaluat* or measure* or test* or screen* or diagnos* or detect* or identif*)).tw.
24 (Personal Wellbeing Index or Personal Well Being Index or PWI*).tw.
25 ("Satisfaction with Life" or "life satisfaction" or SWLS).tw.
26 ("Assessment of Quality of Life" or AQOL*).tw.
27 Health state descriptive system.tw.
28 (ICEpop CAPability measure or ICECAP*).tw.
29 (Sickness impact profile or SIP).tw.
30 (functional limitation* profile or FLP).tw.
31 (((World Health Organi#ation* or WHO) adj2 (Quality of life or QOL)) or WHOQOL*).tw.
32 (Nottingham Health Profile or NHP).tw.
33 (Comprehensive Quality of Life Scale or ComQOL*).tw.
34 (CASP-19* or CASP 19*).tw.
35 (Ageing Well Profile or AWP).tw.
36 (Older People's Quality of Life Questionnaire or OPQOL).tw.
37 (Warwick Edinburgh Mental Well Being Scale or WEMWBS or SWEMWBS).tw.
38 (("Positive and Negative Affect" adj (schedule or scale or survey)) or PANAS).tw.
39 ((Social Production Function or SPF) adj (instrument* or scale*)).tw.
40 (Quality of Life Systemic Inventory or QLSI*).tw.
41 (DTFS scale or DT scale or D-T scale or (delight* adj1 terrible)).tw.
42 Ryff* Psychological Well Being Scale.tw.
43 or/1-42 [quality of life terms]
44 (frail* or sarcop?eni* or prefrailty).tw.
45 cumulative deficit*.tw.

46 phenotype model*.tw.
47 ((Groningen or Tilburg or Edmonton or Fried or Rockwood) adj5 (index* or indicator* or score* or scale* or tool* or test* or model* or phenotype* or criteri* or marker* or method* or instrument* or assess* or exam* or evaluat* or measure* or test* or screen* or diagnos* or detect* or identif*)).tw.
48 (Ensrud or Rothman or Avila Funes).tw.
49 (Gait speed* or walking speed* or grip strength*).tw.
50 hand strength/
51 prisma 7.tw.
52 ("Timed up and go test*" or tugt or gug or "get up and go" or "TUG Test").tw.
53 or/44-52 [frailty terms]
54 aged/
55 aged 80/
56 veteran/
57 geriatrics/
58 (gerontol* or ageing or aging or elder* or geriatric* or senior* or old* age* or late* life or very old or oldest old or old* person or old* people).tw.
59 ((old* adj3 patient*) or (old* adj3 adult*)).tw.
60 or/54-59 [elderly terms]
61 53 and 60 [frailty and elderly terms]
62 frail elderly/
63 61 or 62 [frailty and elderly terms or frail elderly]
64 43 and 63 [quality of life or frail and elderly or frail elderly]
65 limit 64 to yr="1999-current"

CINAHL (Cumulative Index to Nursing and Allied Health Literature)

Search Strategy:

S1 (MH "Quality of Life")
S2 (MH "Health Status")
S3 (MH "Health Status Indicators")
S4 (MH "Surveys")
S5 (MH "Quality-Adjusted Life Years")
S6 TI "health outcome*" OR AB "health outcome*"
S7 TI "quality of life" OR AB "quality of life"
S8 TI (qol or "*qol*") OR AB (qol or "*qol*")
S9 TI (wellbeing or "well being") OR AB (wellbeing or "well being")
S10 TI (HRQL or HRQOL) OR AB (HRQL or HRQOL)
S11 TI "quality adjusted life year*" OR AB "quality adjusted life year*"
S12 TI (QALY* or "*qaly*") OR AB (QALY* or "*qaly*")
S13 TI ("health state" or "health status") OR AB ("health state" or "health status")
S14 TI "health* year* equivalent*" OR AB "health* year* equivalent*"
S15 TI HYE OR AB HYE OR TI ("Medical Outcome* study" or Medical outcome* survey) N3 "36*") or AB("Medical Outcome* study" or Medical outcome* survey) N3 "36*")
S16 TI "life quality" OR AB "life quality"
S17 TI (((short form or shortform) N3 "36*") or (sf36* or sf-36*)) OR AB ((short form or shortform) N3 "36* or (sf36* or sf-36*))
S18 TI (sf12* or sf-12* or sf8* or sf-8* or sf6* or sf-6*) OR AB (sf12* or sf-12* or sf8* or sf-8* or sf6* or sf-6*)
S19 TI (Euroqol or "Euro qol" or EQ-5D* or EQ5D* or EQ-VAS* or EQVAS*) OR AB (Euroqol or "Euro qol" or EQ-5D* or EQ5D* or EQ-VAS* or EQVAS*)

S20 TI ("Quality of Wellbeing Index" or "Quality of Well Being" or QWB*) OR AB ("Quality of Wellbeing Index" or "Quality of Well Being" or QWB*)
S21 TI ("Health utility" or "Health Utility Index" or HUI or SWB or PWB or GWB) OR AB ("Health utility" or "Health Utility Index" or HUI or SWB or PWB or GWB)
S22 TI (RAND-36* or RAND36* or RAND12* or RAND-12* or 36-item RAND or MOSF36* or MOSF-36* or MOS-36*) OR AB (RAND-36* or RAND36* or RAND12* or RAND-12* or 36-item RAND or MOSF36* or MOSF-36* or MOS-36*)
S23 TI (Rosser N3 index* or Rosser N3 indicator* or Rosser N3 score* or Rosser N3 scale* or Rosser N3 tool* or Rosser N3 test* or Rosser N3 model* or Rosser N3 phenotype* or Rosser N3 criteri* or Rosser N3 marker* or Rosser N3 method* or Rosser N3 instrument* or Rosser N3 assess* or Rosser N3 exam* or Rosser N3 evaluat* or Rosser N3 measure* or Rosser N3 test* or Rosser N3 screen* or Rosser N3 diagnos* or Rosser N3 detect* or Rosser N3 identif*) OR AB (Rosser N3 index* or Rosser N3 indicator* or Rosser N3 score* or Rosser N3 scale* or Rosser N3 tool* or Rosser N3 test* or Rosser N3 model* or Rosser N3 phenotype* or Rosser N3 criteri* or Rosser N3 marker* or Rosser N3 method* or Rosser N3 instrument* or Rosser N3 assess* or Rosser N3 exam* or Rosser N3 evaluat* or Rosser N3 measure* or Rosser N3 test* or Rosser N3 screen* or Rosser N3 diagnos* or Rosser N3 detect* or Rosser N3 identif*)
S24 TI (("DTFS scale" or "DT scale" or "D-T scale" or (delight* N terrible)) or AB ("DTFS scale" or "DT scale" or "D-T scale" or (delight* N terrible))
S25 TI (Personal Wellbeing Index or Personal Well Being Index or PWI or PWI-A or "PWI A") OR AB (Personal Wellbeing Index or Personal Well Being Index or PWI or PWI-A or "PWI A")
S26 TI (“Satisfaction with Life" or "life satisfaction" or SWLS) OR AB (“Satisfaction with Life" or "life satisfaction" or SWLS)
S27 TI ("Assessment of Quality of Life" or AQOL*) OR AB ("Assessment of Quality of Life" or AQOL*)
S28 TI "Health state descriptive system" OR AB "Health state descriptive system" or TI ("Quality of Life Systemic Inventory" or QLSI*) or AB ("Quality of Life Systemic Inventory" or QLSI*)
S29 TI ("ICEpop CAPability measure" or ICECAP*) OR AB ("ICEpop CAPability measure" or ICECAP*)
S30 TI "Sickness impact profile" or SIP OR AB "Sickness impact profile" or SIP
S31 TI "functional limitation* profile" or FLP OR AB "functional limitation* profile" or FLP
S32 TI ((World Health Organi#ation* or WHO) N2 (Quality of life or QOL)) or WHOQOL*)) or AB((World Health Organi#ation* or WHO) N2 (Quality of life or QOL))
S33 TI "Nottingham Health Profile" or NHP OR AB "Nottingham Health Profile" or NHP
S34 TI (Comprehensive Quality of Life Scale or ComQOL) OR AB (Comprehensive Quality of Life Scale or ComQOL)
S35 TI CASP-19 OR AB CASP19 or TI (("Social Production Function or SPF)N1 (scale or instrument)) or AB (("Social Production Function or SPF) N1 (scale or instrument))
S36 TI "Ageing Well Profile" or AWP OR AB "Ageing Well Profile" or AWP or TI(("Positive and Negative Affect" N(schedule or scale or survey)) or PANAS)) or TI(("Positive and Negative Affect" N(schedule or scale or survey)) or PANAS))
S37 TI ("Older People's Quality of Life Questionnaire" or OPQOL*) OR AB ("Older People's Quality of Life Questionnaire" or OPQOL*)
S38 TI ("Warwick Edinburgh Mental Well Being Scale" or WEMWBS or SWEMWBS) OR AB ("Warwick Edinburgh Mental Well Being Scale" or WEMWBS or SWEMWBS)
S39 TI "Ryff* Psychological Well Being Scale" or SPF-IL or SPFIL OR AB "Ryff* Psychological Well Being Scale" or SPF-IL or SPFIL
S40 S1 OR S2 OR S3 OR S4 OR S5 OR S6 OR S7 OR S8 OR S9 OR S10 OR S11 OR S12 OR S13 OR S14 OR S15 OR S16 OR S17 OR S18 OR S19 OR S20 OR S21 OR S22 OR S23 OR S24 OR S25 OR S26 OR S27 OR S28 OR S29 OR S30 OR S31 OR S32 OR S33 OR S34 OR S35 OR S36 OR S37 OR S38 OR S39
S41 TI (frail* or sarcop#eni* or prefrail*) OR AB (frail* or sarcop#eni* or prefrail*)
S42 (MH "Sarcopenia")

S43 TI cumulative deficit* OR AB cumulative deficit*
S44 TI phenotype model* OR AB phenotype model*
S45 TI (Groningen N5 index* or Groningen N5 indicator* or Groningen N5 score* or Groningen N5 scale* or Groningen N5 tool* or Groningen N5 test* or Groningen N5 model* or Groningen N5 phenotype* or Groningen N5 criteri* or Groningen N5 marker* or Groningen N5 method* or Groningen N5 instrument* or Groningen N5 assess* or Groningen N5 exam* or Groningen N5 evaluat* or Groningen N5 measure* or Groningen N5 test* or Groningen N5 screen* or Groningen N5 diagnos* or Groningen N5 detect* or Groningen N5 identif*) OR AB (Groningen N5 index* or Groningen N5 indicator* or Groningen N5 score* or Groningen N5 scale* or Groningen N5 tool* or Groningen N5 test* or Groningen N5 model* or Groningen N5 phenotype* or Groningen N5 criteri* or Groningen N5 marker* or Groningen N5 method* or Groningen N5 instrument* or Groningen N5 assess* or Groningen N5 exam* or Groningen N5 evaluat* or Groningen N5 measure* or Groningen N5 test* or Groningen N5 screen* or Groningen N5 diagnos* or Groningen N5 detect* or Groningen N5 identif*)
S46 TI (Tilburg N5 index* or Tilburg N5 indicator* or Tilburg N5 score* or Tilburg N5 scale* or Tilburg N5 tool* or Tilburg N5 test* or Tilburg N5 model* or Tilburg N5 phenotype* or Tilburg N5 criteri* or Tilburg N5 marker* or Tilburg N5 method* or Tilburg N5 instrument* or Tilburg N5 assess* or Tilburg N5 exam* or Tilburg N5 evaluat* or Tilburg N5 measure* or Tilburg N5 test* or Tilburg N5 screen* or Tilburg N5 diagnos* or Tilburg N5 detect* or Tilburg N5 identif*) OR AB (Tilburg N5 index* or Tilburg N5 indicator* or Tilburg N5 score* or Tilburg N5 scale* or Tilburg N5 tool* or Tilburg N5 test* or Tilburg N5 model* or Tilburg N5 phenotype* or Tilburg N5 criteri* or Tilburg N5 marker* or Tilburg N5 method* or Tilburg N5 instrument* or Tilburg N5 assess* or Tilburg N5 exam* or Tilburg N5 evaluat* or Tilburg N5 measure* or Tilburg N5 test* or Tilburg N5 screen* or Tilburg N5 diagnos* or Tilburg N5 detect* or Tilburg N5 identif*)
S47 TI (Edmonton N5 index* or Edmonton N5 indicator* or Edmonton N5 score* or Edmonton N5 scale* or Edmonton N5 tool* or Edmonton N5 test* or Edmonton N5 model* or Edmonton N5 phenotype* or Edmonton N5 criteri* or Edmonton N5 marker* or Edmonton N5 method* or Edmonton N5 instrument* or Edmonton N5 assess* or Edmonton N5 exam* or Edmonton N5 evaluat* or Edmonton N5 measure* or Edmonton N5 test* or Edmonton N5 screen* or Edmonton N5 diagnos* or Edmonton N5 detect* or Edmonton N5 identif*) OR AB (Edmonton N5 index* or Edmonton N5 indicator* or Edmonton N5 score* or Edmonton N5 scale* or Edmonton N5 tool* or Edmonton N5 test* or Edmonton N5 model* or Edmonton N5 phenotype* or Edmonton N5 criteri* or Edmonton N5 marker* or Edmonton N5 method* or Edmonton N5 instrument* or Edmonton N5 assess* or Edmonton N5 exam* or Edmonton N5 evaluat* or Edmonton N5 measure* or Edmonton N5 test* or Edmonton N5 screen* or Edmonton N5 diagnos* or Edmonton N5 detect* or Edmonton N5 identif*)
S48 TI (Fried N5 index* or Fried N5 indicator* or Fried N5 score* or Fried N5 scale* or Fried N5 tool* or Fried N5 test* or Fried N5 model* or Fried N5 phenotype* or Fried N5 criteri* or Fried N5 marker* or Fried N5 method* or Fried N5 instrument* or Fried N5 assess* or Fried N5 exam* or Fried N5 evaluat* or Fried N5 measure* or Fried N5 test* or Fried N5 screen* or Fried N5 diagnos* or Fried N5 detect* or Fried N5 identif*) OR AB (Fried N5 index* or Fried N5 indicator* or Fried N5 score* or Fried N5 scale* or Fried N5 tool* or Fried N5 test* or Fried N5 model* or Fried N5 phenotype* or Fried N5 criteri* or Fried N5 marker* or Fried N5 method* or Fried N5 instrument* or Fried N5 assess* or Fried N5 exam* or Fried N5 evaluat* or Fried N5 measure* or Fried N5 test* or Fried N5 screen* or Fried N5 diagnos* or Fried N5 detect* or Fried N5 identif*)
S49 TI (Rockwood N5 index* or Rockwood N5 indicator* or Rockwood N5 score* or Rockwood N5 scale* or Rockwood N5 tool* or Rockwood N5 test* or Rockwood N5 model* or Rockwood N5 phenotype* or Rockwood N5 criteri* or Rockwood N5 marker* or Rockwood N5 method* or Rockwood N5 instrument* or Rockwood N5 assess* or Rockwood N5 exam* or Rockwood N5 evaluat* or Rockwood N5 measure* or Rockwood

N5 test* or Rockwood N5 screen* or Rockwood N5 diagnos* or Rockwood N5 detect* or Rockwood N5 identif*) OR AB (Rockwood N5 index* or Rockwood N5 indicator* or Rockwood N5 score* or Rockwood N5 scale* or Rockwood N5 tool* or Rockwood N5 test* or Rockwood N5 model* or Rockwood N5 phenotype* or Rockwood N5 criteri* or Rockwood N5 marker* or Rockwood N5 method* or Rockwood N5 instrument* or Rockwood N5 assess* or Rockwood N5 exam* or Rockwood N5 evaluat* or Rockwood N5 measure* or Rockwood N5 test* or Rockwood N5 screen* or Rockwood N5 diagnos* or Rockwood N5 detect* or Rockwood N5 identif*)

S50 TI (Ensrud or Rothman or Avila Funes) OR AB (Ensrud or Rothman or Avila Funes)

S51 TI (Gait speed* or walking speed* or grip strength*) OR AB (Gait speed* or walking speed* or grip strength*)

S52 TI prisma 7 OR AB prisma 7

S53 TI ("Timed up and go test*" or tugt or gug or "get up and go" or "TUG test") OR AB ("Timed up and go test*" or tugt or gug or "get up and go" or "TUG test")

S54 (MH "Grip Strength")

S55 S41 OR S42 OR S43 OR S44 OR S45 OR S46 OR S47 OR S48 OR S49 OR S50 OR S51 OR S52 OR S53 OR S54

S56 (MH "Aged")

S57 (MH "Aged, 80 and Over")

S58 (MH "Veterans")

S59 (MH "Geriatrics")

S60 TI (gerontol* or ageing or aging or elder* or geriatric* or senior* or old* age* or late* life or very old or oldest old or old* person or old* people) OR AB (gerontol* or ageing or aging or elder* or geriatric* or senior* or old* age* or late* life or very old or oldest old or old* person or old* people)

S61 TI ((old* N3 patient*) or (old* N3 adult*)) OR AB ((old* N3 patient*) or (old* N3 adult*))

S62 S56 OR S57 OR S58 OR S59 OR S60 OR S61

S63 S55 AND S62

S64 (MH "Frail Elderly")

S65 S63 OR S64

S66 S40 AND S65

S67 TI (observational study or observational studies) OR AB (observational study or observational studies)

S68 TI (cohort study or cohort studies) OR AB (cohort study or cohort studies)

S69 (MH "Cross Sectional Studies")

S70 (MH "Nonconcurrent Prospective Studies")

S71 (MH "Correlational Studies")

S72 (MH "Case Control Studies+")

S73 (MH "Prospective Studies")

S74 S67 OR S68 OR S69 OR S70 OR S71 OR S72 OR S73

S75 S66 AND S74. Limiters - Published Date: 19990101-20170131

Cochrane Library

Search Strategy:

#1 (Quality of Life Systemic Inventory or QLSI*):ti,ab,kw

#2 ("social production function instrument" or "social production function scale"):ti,ab,kw

#3 (MOSF36* or MOSF-36* or MOS-36*):ti,ab,kw

#4 (RAND-36* or RAND36* or RAND12* or RAND-12* or 36-item RAND):ti,ab,kw

#5 ("Positive and Negative Affect" near/1 (schedule or scale or survey) or PANAS):ti,ab,kw

#6 (DTFS scale or DT scale or D-T scale or (delight* near/1 terrible)):ti,ab,kw

#7 MeSH descriptor: [Quality of Life] this term only
#8 MeSH descriptor: [Health Status] this term only
#9 MeSH descriptor: [Health Status Indicators] this term only
#10 MeSH descriptor: [Health Surveys] this term only
#11 MeSH descriptor: [Quality-Adjusted Life Years] this term only
#12 quality of life:ti,ab,kw (Word variations have been searched)
#13 (QOL or "*qol*"):ti,ab,kw (Word variations have been searched)
#14 (wellbeing or well being):ti,ab,kw (Word variations have been searched)
#15 (HRQL or HRQOL):ti,ab,kw (Word variations have been searched)
#16 quality adjusted life year*:ti,ab,kw (Word variations have been searched)
#17 (QALY* or "*qaly*"):ti,ab,kw (Word variations have been searched)
#18 Health state:ti,ab,kw (Word variations have been searched)
#19 health status:ti,ab,kw (Word variations have been searched)
#20 health* year* equivalent*:ti,ab,kw (Word variations have been searched)
#21 life quality:ti,ab,kw (Word variations have been searched)
#22 (((short form or shortform) near/3 "36*") or (sf36* or sf-36*)):ti,ab,kw
#23 (sf12* or sf-12* or sf8* or sf-8* or sf6* or sf-6*):ti,ab,kw
#24 (Euroqol or Euro qol or EQ-5D* or EQ5D* or EQ-VAS* or EQVAS*):ti,ab,kw
#25 (Quality of Wellbeing Index or Quality of Well Being or QWB or QWB-SA):ti,ab,kw (Word variations have been searched)
#26 (Health utility or Health Utility Index or HUI):ti,ab,kw (Word variations have been searched)
#27 (HYE or HYES):ti,ab,kw (Word variations have been searched)
#28 (SWB or PWB or GWB):ti,ab,kw (Word variations have been searched)
#29 (Medical Outcome* near/1 (study or survey) near/3 36*):ti,ab,kw
#30 (Personal Wellbeing Index or Personal Well Being Index or PWI*):ti,ab,kw
#31 ("Satisfaction with Life" or "life satisfaction" or SWLS):ti,ab,kw
#32 (Assessment of Quality of Life or AQOL*):ti,ab,kw (Word variations have been searched)
#33 Health state descriptive system:ti,ab,kw (Word variations have been searched)
#34 (ICEpop CAPability measure or ICECAP*):ti,ab,kw
#35 (Sickness impact profile or SIP):ti,ab,kw (Word variations have been searched)
#36 (functional limitation* profile or FLP):ti,ab,kw (Word variations have been searched)
#37 (((World Health Organi?ation* or WHO) near/2 (Quality of life or QOL)) or WHOQOL*):ti,ab,kw
#38 (Nottingham Health Profile or NHP):ti,ab,kw (Word variations have been searched)
#39 (Comprehensive Quality of Life Scale or ComQOL*):ti,ab,kw
#40 (CASP19* or CASP-19*):ti,ab,kw
#41 (Ageing Well Profile or AWP):ti,ab,kw (Word variations have been searched)
#42 (Older People's Quality of Life Questionnaire or OPQOL):ti,ab,kw (Word variations have been searched)
#43 (Warwick Edinburgh Mental Well Being Scale or WEMWBS or SWEMWBS):ti,ab,kw (Word variations have been searched)
#44 Ryff* Psychological Well Being Scale:ti,ab,kw (Word variations have been searched)
#45 health outcome*:ti,ab,kw (Word variations have been searched)
#46 (Rosser near/3 (index* or indicator* or score* or scale* or tool* or test* or model* or phenotype* or criteri* or marker* or method* or instrument* or assess* or exam* or evaluat* or measure* or test* or screen* or diagnos* or detect* or identif*)):ti,ab,kw (Word variations have been searched)
#47 {or #1-#46}
#48 (frail* or sarcop?eni* or prefrailty):ti,ab,kw (Word variations have been searched)
#49 MeSH descriptor: [Sarcopenia] explode all trees
#50 cumulative deficit*:ti,ab,kw (Word variations have been searched)
#51 phenotype model*:ti,ab,kw (Word variations have been searched)

#52 ((Groningen or Tilburg or Edmonton or Fried or Rockwood) near/5 (index* or indicator* or score* or scale* or tool* or test* or model* or phenotype* or criteri* or marker* or method* or instrument* or assess* or exam* or evaluat* or measure* or test* or screen* or diagnos* or detect* or identif*)):ti,ab,kw (Word variations have been searched)
#53 (Ensrud or Rothman or Avila Funes):ti,ab,kw (Word variations have been searched)
#54 (Gait speed* or walking speed* or grip strength*):ti,ab,kw (Word variations have been searched)
#55 MeSH descriptor: [Hand Strength] explode all trees
#56 prisma 7:ti,ab,kw (Word variations have been searched)
#57 ("Timed up and go test*" or tugt or gug or "get up and go" or "TUG Test"):ti,ab,kw (Word variations have been searched)
#58 {or #48-#57}
#59 MeSH descriptor: [Aged] this term only
#60 MeSH descriptor: [Aged, 80 and over] this term only
#61 MeSH descriptor: [Veterans] this term only
#62 MeSH descriptor: [Geriatrics] this term only
#63 (gerontol* or ageing or aging or elder* or geriatric* or senior* or old* age* or late* life or very old or oldest old or old* person or old* people):ti,ab,kw (Word variations have been searched)
#64 ((old*near/3 patient*) or (old* near/3 adult*)):ti,ab,kw (Word variations have been searched)
#65 {or #59-#64}
#66 #58 and #65
#67 MeSH descriptor: [Frail Elderly] this term only
#68 #66 or #67
#69 #47 and #57 Publication Year from 1999 to 2017

Ovid MEDLINE

Search Strategy:

----

1 "quality of life"/
2 health status/
3 health status indicator/
4 health surveys/
5 quality adjusted life years/
6 health outcome*.tw.
7 quality of life.tw.
8 (QOL or "*qol*").tw.
9 (wellbeing or well being).tw.
10 (HRQL or HRQOL).tw.
11 quality adjusted life year*.tw.
12 (QALY* or "*qaly*").tw.
13 Health state.tw.
14 health status.tw.
15 health* year* equivalent*.tw.
16 life quality.tw.
17 (((short form or shortform) adj3 "36*") or (sf36* or sf-36*)).tw.
18 (sf12* or sf-12* or sf8* or sf-8* or sf6* or sf-6*).tw.
19 (Euroqol or Euro qol or EQ-5D* or EQ5D* or EQ-VAS* or EQVAS*).tw.
20 (Quality of Wellbeing Index or Quality of Well Being or QWB*).tw.
21 (RAND-36* or RAND36* or RAND12* or RAND-12* or 36-item RAND).tw.

22 (MOSF36* or MOSF-36* or MOS-36*).tw.
23 (Health utility or Health Utility Index or HUI).tw.
24 (HYE or HYES).tw.
25 (SWB or PWB or GWB).tw.
26 (Medical Outcome* adj (study or survey) adj3 36*).tw.
27 (Rosser adj3 (index* or indicator* or score* or scale* or tool* or test* or model* or phenotype* or criteri* or marker* or method* or instrument* or assess* or exam* or evaluat* or measure* or test* or screen* or diagnos* or detect* or identif*)).tw.
28 (Personal Wellbeing Index or Personal Well Being Index or PWI*).tw.
29 ("Satisfaction with Life" or "life satisfaction" or SWLS).tw.
30 (Assessment of Quality of Life or AQOL*).tw.
31 Health state descriptive system.tw.
32 (ICEpop CAPability measure or ICECAP*).tw.
33 (Sickness impact profile or SIP).tw.
34 (functional limitation* profile or FLP).tw.
35 (((World Health Organi#ation* or WHO) adj2 (Quality of life or QOL)) or WHOQOL*).tw.
36 (Nottingham Health Profile or NHP).tw.
37 (Comprehensive Quality of Life Scale or ComQOL*).tw.
38 (CASP19* or CASP-19*).tw.
39 (Ageing Well Profile or AWP).tw.
40 (Older People's Quality of Life Questionnaire or OPQOL).tw.
41 (Warwick Edinburgh Mental Well Being Scale or WEMWBS or SWEMWBS).tw.
42 (DTFS scale or DT scale or D-T scale or (delight* adj1 terrible)).tw.
43 (("Positive and Negative Affect" adj (schedule or scale or survey)) or PANAS).tw.
44 ((Social Production Function or SPF) adj (instrument* or scale*)).tw.
45 (Quality of Life Systemic Inventory or QLSI*).tw.
46 Ryff* Psychological Well Being Scale.tw.
47 or/1-46 [quality of life terms]
48 (frail* or sarcop?eni* or prefrailty).tw.
49 sarcopenia/
50 cumulative deficit*.tw.
51 phenotype model*.tw.
52 ((Groningen or Tilburg or Edmonton or Fried or Rockwood) adj5 (index* or indicator* or score* or scale* or tool* or test* or model* or phenotype* or criteri* or marker* or method* or instrument* or assess* or exam* or evaluat* or measure* or test* or screen* or diagnos* or detect* or identif*)).tw.
53 (Ensrud or Rothman or Avila Funes).tw.
54 (Gait speed* or walking speed* or grip strength*).tw.
55 exp hand strength/
56 prisma 7.tw.
57 ("Timed up and go test*" or tugt or gug or "get up and go" or "TUG Test").tw.
58 or/48-57 [frailty terms]
59 aged/
60 "Aged, 80 and over"/
61 veteran/
62 geriatrics/
63 (gerontol* or ageing or aging or elder* or geriatric* or senior* or old* age* or late* life or very old or oldest old or old* person or old* people).tw.
64 ((old* adj3 patient*) or (old* adj3 adult*)).tw.
65 or/59-64 [elderly terms]
66 58 and 65 [frailty and elderly terms]
67 frail elderly/
68 66 or 67 [frailty and elderly terms or frail elderly]
69 47 and 68 [quality of life and frail and elderly or frail elderly]

70 Epidemiologic Studies/
71 exp Case-Control Studies/
72 exp Cohort Studies/
73 case control.tw.
74 (cohort adj (study or studies)).tw.
75 cohort analy*.tw.
76 (Follow up adj (study or studies)).tw.
77 Longitudinal.tw.
78 cohort analysis/
79 Retrospective.tw.
80 Cross sectional.tw.
81 Cross-Sectional Studies/
82 or/70-81 [SIGN observational studies filter]
83 69 and 82 [quality of llife and frail and elderly or frail elderly and cohort or cross sectional]
84 (ANIMALS not HUMANS).sh.
85 83 not 84 [human only studies]
86 limit 85 to yr="1999-current"
87 remove duplicates from 86

PsycINFO

Search Strategy:

----

1 "quality of life"/
2 health status/
3 health survey*.tw.
4 health outcome*.tw.
5 quality of life.tw.
6 (QOL or "*qol*").tw.
7 (wellbeing or well being).tw.
8 (HRQL or HRQOL).tw.
9 quality adjusted life year*.tw.
10 (QALY* or "*qaly*").tw.
11 Health state.tw.
12 health status.tw.
13 health* year* equivalent*.tw.
14 life quality.tw.
15 (sf12* or sf-12* or sf8* or sf-8* or sf6* or sf-6*).tw.
16 (((short form or shortform) adj3 "36*") or (sf36* or sf-36*)).tw.
17 (Euroqol or Euro qol or EQ-5D* or EQ5D* or EQ-VAS* or EQVAS*).tw.
18 (MOSF36* or MOSF-36* or MOS-36*).tw.
19 (RAND-36* or RAND36* or RAND12* or RAND-12* or 36-item RAND).tw.
20 (Quality of Wellbeing Index or Quality of Well Being or QWB*).tw.
21 (Health utility or Health Utility Index or HUI).tw.
22 (HYE or HYES).tw.
23 (SWB or PWB or GWB).tw.
24 (Medical Outcome* adj (study or survey) adj3 36*).tw.
25 (Rosser adj3 (index* or indicator* or score* or scale* or tool* or test* or model* or phenotype* or criteri* or marker* or method* or instrument* or assess* or exam* or evaluat* or measure* or test* or screen* or diagnos* or detect* or identif*)).tw.
26 (Personal Wellbeing Index or Personal Well Being Index or PWI*).tw.
27 ("Satisfaction with Life" or "life satisfaction" or SWLS).tw.
28 (Assessment of Quality of Life or AQOL*).tw.

29 Health state descriptive system.tw.
30 (ICEpop CAPability measure or ICECAP or ICECAP-A or ICECAP-O).tw.
31 (Sickness impact profile or SIP).tw.
32 (functional limitation* profile or FLP).tw.
33 (((World Health Organi#ation* or WHO) adj2 (Quality of life or QOL)) or WHOQOL*).tw.
34 (Nottingham Health Profile or NHP).tw.
35 (Comprehensive Quality of Life Scale or ComQOL*).tw.
36 (CASP19* or CASP-19*).tw.
37 (Ageing Well Profile or AWP).tw.
38 (Older People's Quality of Life Questionnaire or OPQOL).tw.
39 (("Positive and Negative Affect" adj (schedule or scale or survey)) or PANAS).tw.
40 (DTFS scale or DT scale or D-T scale or (delight* adj1 terrible)).tw.
41 ((Social Production Function or SPF) adj (instrument* or scale*)).tw.
42 (Warwick Edinburgh Mental Well Being Scale or WEMWBS or SWEMWBS).tw.
43 (Quality of Life Systemic Inventory or QLSI*).tw.
44 Ryff* Psychological Well Being Scale.tw.
45 or/1-44 [quality of life terms]
46 (frail* or sarcop?eni* or prefrailty).tw.
47 cumulative deficit*.tw.
48 phenotype model*.tw.
49 ((Groningen or Tilburg or Edmonton or Fried or Rockwood) adj5 (index* or indicator* or score* or scale* or tool* or test* or model* or phenotype* or criteri* or marker* or method* or instrument* or assess* or exam* or evaluat* or measure* or test* or screen* or diagnos* or detect* or identif*)).tw.
50 (Ensrud or Rothman or Avila Funes).tw.
51 (Gait speed* or walking speed* or grip strength*).tw.
52 hand strength.tw.
53 prisma 7.tw.
54 ("Timed up and go test*" or tugt or gug or "get up and go" or "TUG Test").tw.
55 or/46-54 [frailty terms]
56 exp aging/
57 veteran/
58 geriatrics/
59 geriatric patients/
60 (gerontol* or ageing or aging or elder* or geriatric* or senior* or old* age* or late* life or very old or oldest old or old* person or old* people).tw.
61 ((old* adj3 patient*) or (old* adj3 adult*)).tw.
62 or/56-61 [aged terms]
63 55 and 62 [frailty and aged terms]
64 63 and 45 [quality of life and frailty and aged terms]
65 limit 64 to yr="1999-current"

Web of Science

Search Strategy:
----

1 TOPIC: (frail* or sarcop?eni* or prefrailty)
2 TOPIC: (cumulative deficit*)
3 TOPIC: (cumulative deficit* or phenotype model*)
4 TOPIC: ((((Groningen or Tilburg or Edmonton or Prisma* or Fried or Rockwood) NEAR/5 (index* or indicator* or score* or scale* or tool* or test* or model* or phenotype* or criteri* or marker* or method* or instrument* or assess* or exam* or evaluat* or measure* or test* or screen* or diagnos* or detect* or identif*))))

5 TOPIC: (((Ensrud or Rothman or "Avila Funes")))
6 TOPIC: ((("Gait speed*" or "walking speed*" or "grip strength*")))
7 TOPIC: ((("prisma 7")))
8 TOPIC: ("Timed up and go test*" or tugt or gug or "get up and go" or "TUG Test")
9 TOPIC: (old* Near/3 patient* or old* Near/3 adult*)
10 TOPIC: (gerontol* OR ageing OR aging OR elder* OR geriatric* OR senior* OR "old age*" OR older OR "late* life" OR "very old" OR "oldest old")
11 #8 OR #7 OR #6 OR #5 OR #4 OR #3 OR #2 OR #1
12 #10 AND #9
13 TOPIC: (("quality of life or health survey"* or "health outcome*" or QOL or "*qol*" or wellbeing or "well being" or HRQL or HRQOL or "quality adjusted life year*" or QALY* or "*qaly*" or "Health state" or "health status" or "health* year* equivalent*" or "life quality"))
14 TS=((short form NEAR/3 "36" or shortform NEAR/3 "36" or f36* or sf-36*))
15 TOPIC: ((sf12* or sf-12* or sf8* or sf-8* or sf6* or sf-6*))
16 TOPIC: ((Euroqol or "Euro qol" or EQ-5D* or EQ5D* or EQ-VAS* or EQVAS*))
17 TOPIC: (("Quality of Wellbeing Index" or "Quality of Well Being" or QWB*))
18 TOPIC: ((RAND-36* or RAND36* or RAND12* or RAND-12* or 36-item RAND))
19 TOPIC: ((MOSF36* or MOSF-36* or MOS-36*))
20 TOPIC: (("Health utility" or HUI))
21 TOPIC: ((HYE or HYES))
22 TOPIC: ((SWB or PWB or GWB))
23 TOPIC: (("Medical Outcome* study" or "Medical outcome* survey") Near/3 "36")
24 TOPIC: ((Rosser NEAR/3 (index* or indicator* or score* or scale* or tool* or test* or model* or phenotype* or criteri* or marker* or method* or instrument* or assess* or exam* or evaluat* or measure* or test* or screen* or diagnos* or detect* or identif*)))

25 TOPIC: ("Personal Wellbeing Index" or "Personal Well Being Index" or PWI*)
26 TOPIC: ("Satisfaction with Life" or "life satisfaction" or SWLS)
27 TOPIC: (("Assessment of Quality of Life" or AQOL))
28 TOPIC: ("Health state descriptive system")
29 TOPIC: ("ICEpop CAPability measure" or ICECAP*)
30 TOPIC: ("Sickness impact profile" or SIP)
31 TOPIC: ("functional limitation* profile" or FLP)
32 TOPIC: ((("World Health Organi$ation*" or WHO) NEAR/2 ("Quality of life" or QOL)))
33 TOPIC: (("Nottingham Health Profile" or NHP))
34 TOPIC: ("comprehensive Quality of Life Scale" or ComQOL*)
35 TS=(CASP19* or CASP-19*) or TS=("Ryff* Psychological Well Being Scale") or TS=("Quality of Life Systemic Inventory" or QLSI*)
36 TS=("Ageing Well Profile" or AWP) or TS=(("Social Production Function" or SPF) NEAR/2 (instrument* or scale))
37 TOPIC: ("Older People's Quality of Life Questionnaire" or OPQOL)
38 TOPIC: ("Warwick Edinburgh Mental Well Being Scale" or WEMWBS or SWEMWBS)
39 TOPIC: ("DTFS scale" or "DT scale" or "D-T scale" or (delight* NEAR/1 terrible))
40 TOPIC: ("Positive and Negative Affect" NEAR (schedule or scale or survey) or PANAS)
41 #40 OR #39 OR #38 OR #37 OR #36 OR #35 OR #34 OR #33 OR #32 OR #31 OR #30 OR #29 OR #28 OR #27 OR #26 OR #25 OR #24 OR #23 OR #22 OR #21 OR #20 OR #19 OR #18 OR #17 OR #16 OR #15 OR #14 OR #13
42 #41 AND #12. Timespan=1999-2017
