# Supplementary material for: Quality of life is substantially worse for community-dwelling older people living with frailty: systematic review and meta-analysis
Source: Qual Life Res. 2019 Mar 14;28(8):2041–56. doi: 10.1007/s11136-019-02149-1 (PMC6620381; doi:10.1007/s11136-019-02149-1)
Supplement: Supplementary file 2 — Supplementary material 2 (PDF 23 KB) [file 11136_2019_2149_MOESM2_ESM.pdf]

Online Resource 2 for “Quality of life is substantially worse for community-dwelling older people living with frailty: systematic review and meta-analysis”. *Quality of Life Research*. Crocker TF*, Brown L, Clegg A, Farley K, Franklin M, Simpkins S, Young J. * Bradford Teaching Hospitals NHS Foundation Trust; tom.crocker@bthft.nhs.uk

**Study name**			
**Reviewer**			
**Papers**			
**Extra data provided by author**			
**Title of trial (if known)**			
**Aims of study**			
**Links to other papers**			
**Study type**			
**Country**			
**Setting (e.g. urban)**			
**Characterisation of participants**			
**Years when reported data collected**			
**Age: mean (SD)**			
**% female**			
**Total in analysis**			
**Frailty ascertainment**			
**Quality of life instruments**			
**Does the paper describe QOL instruments as measuring quality of life /well-being / life satisfaction?**			
categories for N and Means	Fit (or robust) [†]	Pre-frail (or mild frailty) ‡	Frail (or moderate-severe frailty) §
**N: Not frail (Fit); pre frail; frail: number (% out of total population)**			
**Quality of life data by frailty status. Report means, SDs, SEs, or median and interquartile or other descriptors if used**			
**Reported associations between frailty and quality of life**			

statistical methods used to assess the data (e.g. hypothesis tests such as t-test of chi-test for assessing mean difference, or regression analysis and the type of model used).		
Covariates adjusted for (if any)		
Authors conclusions (just a couple of lines to summarise)		
Additional information		
Notable exclusions		
Quality assessment	Decision	Justification
Representativeness of the sample:		
Sample size:		
Non-respondents:		
Ascertainment of frailty:		
The subjects in different outcome groups are comparable, based on the study design or analysis. Confounding factors are controlled.		
Assessment of the outcome:		
Statistical test:		
