# Supplementary material for: Quality of life is substantially worse for community-dwelling older people living with frailty: systematic review and meta-analysis
Source: Qual Life Res. 2019 Mar 14;28(8):2041–56. doi: 10.1007/s11136-019-02149-1 (PMC6620381; doi:10.1007/s11136-019-02149-1)
Supplement: Supplementary file 3 — Supplementary material 3 (PDF 140 KB) [file 11136_2019_2149_MOESM3_ESM.pdf]

Online Resource 3 for "Quality of life is substantially worse for community-dwelling older people living with frailty: systematic review and meta-analysis". *Quality of Life Research*. Crocker TF*, Brown L, Clegg A, Farley K, Franklin M, Simpkins S, Young J. * Bradford Teaching Hospitals NHS Foundation Trust; tom.crocker@bthft.nhs.uk

Aids to interpretation of differences

Rules of thumb for standardized mean differences

Standardised mean differences developed from the original rules of thumb by Cohen (for small medium and large differences) . The rules of thumb are defined as point values, e.g. small = 0.2, which we have conservatively taken as the lower bounds for bins.

Bin	Lower boundary (d ≥)	Upper boundary (d <)
**very small**	.01	.2
**small**	.2	.5
**medium**	.5	.8
**large**	.8	1.2
**very large**	1.2	2.0
**huge**	2.0	-

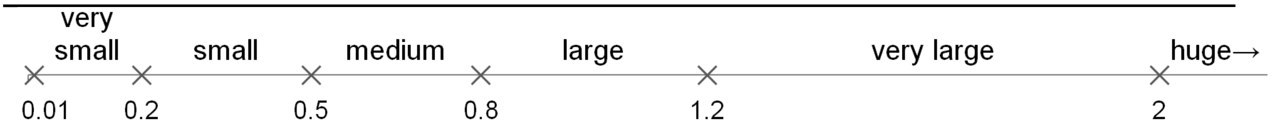

Meaningful differences in general populations

Medical Outcomes Study 36-item Short-Form health survey (SF-36)

Mean differences in each sub-scale of the SF-36 between patients with minor vs. serious medical conditions in the Medical Outcomes Study (n=806)

Sub-scale	Medical condition		Mean difference
	Minor	Serious	
Physical functioning (PF)	80.5	57.4	23.2
Role-physical (RP)	70.3	43.9	26.4
Bodily pain (BP)	76.1	65.1	11.0
Mental Health (MH)	82.5	77.6	4.9
Role-emotional (RE)	84.3	76.2	8.1
Social functioning (SF)	91.6	80.0	11.6
Vitality (VT)	62.0	47.8	14.2
General health perceptions (GH)	67.0	49.1	17.9

Mean differences in summary scales of the SF-36 between the 25th and 75th percentile from a general sample (n=8,207)

Component scale	25th percentile	75th percentile	Mean difference
Physical component summary scale (PCS)	47.1	56.1	9.1
Mental component	46.1	57.0	10.8

summary scale (MCS)			

World Health Organisation Quality of Life short-form instrument (WHOQOL-BREF)

Mean differences between "well" and "sick" groups from a broad sample (n = 11,380) following a previous example

Domain	Well	Sick	Mean difference
Physical	71.3	56.9	14.4
Psychological	67.5	60.6	6.9
Social	67.5	62.5	5.0
Environment	63.1	61.3	1.9

References

[1] Sawilowsky SS. New Effect Size Rules of Thumb. *Journal of Modern Applied Statistical Methods*. 2009;8(2):Article 26. doi: 10.22237/jmasm/1257035100.

[2] McHorney CA, Ware JE, Raczek AE. The MOS 36-Item Short-Form Health Survey (SF-36): II. Psychometric and clinical tests of validity in measuring physical and mental health constructs. *Medical Care*. 1993;31(3):247-63.

[3] Jenkinson C. The SF-36 Physical and Mental Health Summary Measures: An Example of How to Interpret Scores. *Journal of Health Services Research & Policy*. 1998;3(2):92-6. doi: 10.1177/135581969800300206.

[4] Skevington SM, Lotfy M, O'Connell KA. The World Health Organization's WHOQOL-BREF quality of life assessment: psychometric properties and results of the international field trial. A report from the WHOQOL group. *Quality of Life Research*. 2004;13(2):299-310. doi: 10.1023/B:QURE.0000018486.91360.00.

[5] Crocker TF, Smith JK, Skevington SM. Family and professionals underestimate quality of life across diverse cultures and health conditions: systematic review. *Journal of Clinical Epidemiology*. 2015;68(5):584-95. doi: 10.1016/j.jclinepi.2014.12.007.
