# Supplementary material for: Quality of life is substantially worse for community-dwelling older people living with frailty: systematic review and meta-analysis
Source: Qual Life Res. 2019 Mar 14;28(8):2041–56. doi: 10.1007/s11136-019-02149-1 (PMC6620381; doi:10.1007/s11136-019-02149-1)
Supplement: Supplementary file 4 — Supplementary material 4 (PDF 208 KB) [file 11136_2019_2149_MOESM4_ESM.pdf]

Online Resource 4 for “Quality of life is substantially worse for community-dwelling older people living with frailty: systematic review and meta-analysis”. *Quality of Life Research*. Crocker TF*, Brown L, Clegg A, Farley K, Franklin M, Simpkins S, Young J. * Bradford Teaching Hospitals NHS Foundation Trust; tom.crocker@bthft.nhs.uk

Details of included quality of life instruments

Measure name	Construct according to developer(s)	Number of items, direction† and item-scaling	Summary scores (operation‡: range§)	Sub-scales (operation‡: range§)	Notes
CASP-19	QOL / needs satisfaction / well-being	19 ↑<br>Likert 0 to 3	Total (∑: 0 to 57)	Control (∑: 0 to 12)<br>ability to intervene in one's environment<br>Autonomy (∑: 0 to 15)<br>freedom from unwanted interference<br>Self-realization (∑: 0 to 15)<br>actively being and growing<br>Pleasure (∑: 0 to 15)<br>happiness	Following Steptoe et al., included study Gale 2014 combined the control, autonomy and self-realization scales of the CASP-19 as a “eudaimonic” scale, and renamed the pleasure scale “hedonic”.
EQ-VAS	Self-rated health	1 ↑<br>VAS 0 to 100	Score (0 to 100)	*None: one item only.*	The EQ-VAS is part of the EuroQol 5-Dimension (EQ-5D) health questionnaire (not described in this document)
EUROHIS-QOL 8	QOL	8 ↑<br>Likert 1 to 5	Score ($\bar{x}$: 1 to 5)	*None*	The eight items of the EUROHIS-QOL 8 are drawn from the 26 items of the WHOQOL-BREF, including the overall assessment of QOL, overall assessment of health, two physical, one psychological, one social and two environment domain items.
Life satisfaction (Pinto 2016)	Life satisfaction	6 ↑<br>Likert 1 to 4	Score (∑: 6 to 24)	*None*	This unreferenced instrument has questions about satisfaction with life overall, friendship, environment, ability to do daily tasks, health services and means of transportation
Life satisfaction in 13 unspecified domains (Yang 2016)	Life satisfaction	13 ↑<br>Likert 1 to 5	Score (∑: 13 to 65)	*None*	This unreferenced instrument has questions on 13 unspecified domains. Sample items included “How satisfied are youwith your health status?” and “How satisfied are you with yourfamily relationships?”

Measure name	Construct according to developer(s)	Number of items, direction† and item-scaling	Summary scores (operation‡: range§)	Sub-scales (operation‡: range§)	Notes
Life-Satisfaction Terrible-Delightful scale (St John 2013)	Individual well-being / Life satisfaction	10 ↑<br>Likert 1 to 7	Health (1 to 7)<br>Finances (1 to 7)<br>Family relations (1 to 7)<br>Friendships (1 to 7)<br>Housing (1 to 7)<br>Recreation activity (1 to 7)<br>Spiritual fulfillment (1 to 7)<br>Self-esteem (1 to 7)<br>Transportation (1 to 7)<br>Life overall (1 to 7)	*None: each item is scored separately*	The format is based on the Delighted-Terrible scale but the items have been changed.
OPQOL	QOL in older age	35 ↑<br>Likert 1 to 5	Total (∑: 35 to 175)	Life overall (∑: 4 to 20)<br>Health (∑: 4 to 20)<br>Social relationships and participation (∑: 5 to 25)<br>Independence, control over life, freedom (∑: 4 to 20)<br>Area: home and neighbourhood (∑: 4 to 20)<br>Psychological and emotional well-being (∑: 4 to 20)<br>Financial circumstances (∑: 4 to 20)<br>Religion/culture (∑: 6 to 30)	
Quality of life overall (Ament 2014)	QOL	1 ↓<br>Five options coded: excellent, very good or good = 0; moderate or bad = 1	Category (0 / 1)	*None: one item only*	This unreferenced instrument has a single question about quality of life overall.

Measure name	Construct according to developer(s)	Number of items, direction† and item-scaling	Summary scores (operation‡: range§)	Sub-scales (operation‡: range§)	Notes
Ryff's Psychological Well-Being scale (18 item version)	Psychological well-being / general well-being	18 ↑<br>Likert 1 to 6	Total (∑: 18 to 108)	Autonomy (∑: 3 to 18)<br>independence and self-determination<br>Environmental mastery (∑: 3 to 18)<br>ability to manage one's life<br>Personal growth (∑: 3 to 18)<br>being open to new experiences<br>Positive relations with others (∑: 3 to 18)<br>having satisfying high quality relationships<br>Purpose in life (∑: 3 to 18)<br>believing that one's life is meaningful<br>Self-acceptance (∑: 3 to 18)<br>positive attitude towards oneself and one's past life	In included report Andrew 2012 the scores are reversed so that greater scores equal worse well-being, but raw scores are not reported in this review as data was not presented in a suitable format.
SF-12	Health status / Health-related QOL	12 ↑<br>Between two and six response options. Each response has a particular weight for each summary score.	Physical Component Summary (PCS-12) (f: 10 to 70)<br>Mental Component Summary (MCS-12) (f: 6 to 72)	*None*	The PCS-12 and MCS-12 are both functions of all 12 items and a constant. They are designed to replicate scores from the SF-36 PCS and MCS based on regression analysis.

Measure name	Construct according to developer(s)	Number of items, direction† and item-scaling	Summary scores (operation‡: range§)	Sub-scales (operation‡: range§)	Notes
SF-36	Health status / Health-related QOL	36 ↑<br>Between two and six response options. 2 items are scored with unequal intervals	Physical Component Summary (PCS) (f: 2 to 76)<br>Mental Component Summary (MCS) (f: -1 to 81)	Physical functioning (L: 0 to 100)<br>limitations in physical activities because of health problems<br>Role—Physical (L: 0 to 100)<br>limitations in usual role activities because of physical health problems<br>Bodily pain (L: 0 to 100)<br>Severity and interference of bodily pain<br>General health(L: 0 to 100)<br>general health perceptions<br>Vitality (L: 0 to 100)<br>energy and fatigue / subjective well-being<br>Social functioning (L: 0 to 100)<br>limitations in social activities because of physical or emotional problems<br>Role—Emotional (L: 0 to 100)<br>limitations in usual role activities because of emotional problems<br>Mental health (L: 0 to 100)<br>psychological distress and well-being	The PCS and MCS are both functions of all eight sub-scales based on principal component analysis, and are transformed to yield means of 50 and standard deviations of 10 for a general national population.<br>The SF-36 includes an additional Health Transition item that does not contribute to the scales or summary scores and is not presented in the cited studies.
SWLS	Global life satisfaction	5 ↑<br>Likert 1 to 7	Score ($\sum$: 5 to 35)	*None*	Five global life satisfaction items (life ideal, excellent conditions, satisfied with life, have what I want, would change nothing)

Measure name	Construct according to developer(s)	Number of items, direction† and item-scaling	Summary scores (operation‡: range§)	Sub-scales (operation‡: range§)	Notes
WHOQOL-BREF	QOL	26 ↑<br>Likert 1 to 5	Physical (L: 0 to 100)<br>personal evaluation of physical health<br>Psychological (L: 0 to 100)<br>personal evaluation of psychological state<br>Social (L: 0 to 100)<br>satisfaction with social relationships<br>Enviroment (L: 0 to 100)<br>satisfaction with salient features of the environment (healthiness and safety of environment, living place, finances, information, leisure, transport and access to healthcare)	*None*	The four summary scores (domains) are calculated from 24 items, each of which represents one of the 24 facets of WHOQOL-100 (not described in this document). Two additional items, an overall assessment of QOL and an overall assessment of health are part of the WHOQOL-BREF but do not form part of the domains and are not reported in the included studies.
WHOQOL-OLD	QOL of older adults	24 ↑<br>Likert 1 to 5	Total (x: 1 to 5)	Sensory abilities (x: 1 to 5)<br>sensory functioning and the impact of loss of sensory abilities on QOL<br>Autonomy (x: 1 to 5)<br>ability to live autonomously and to take own decisions<br>Past, present and future activities (x: 1 to 5)<br>satisfaction about achievements in life and things to look forward to<br>Social Participation (x: 1 to 5)<br>participation in activities of daily living, especially in the community<br>Death and Dying (x: 1 to 5)<br>concerns, worries, and fears about death and dying<br>Intimacy (x: 1 to 5)<br>ability to have personal and intimate relationships	The six sub-scales (facets) are additional to the 24 facets of the WHOQOL-100 (not described in this document).

†	Direction after any reversing of item scores: ↑ Greater scores indicate better QOL; ↓ Lesser scores indicate better QOL.
‡	Operation performed on item scores to produce scale score. $\sum$: Sum of items; x: Mean of items; L: Mean of a linear transformation of the items; f: Other function (see notes); absent in the case of single item scales
§	Theoretical score range
VAS	Visual-analogue scale

References

[1] Hyde M, Wiggins RD, Higgs P, Blane DB. A measure of quality of life in early old age: The theory, development and properties of a needs satisfaction model (CASP-19). *Aging & Mental Health*. 2003;7(3):186-94. doi: 10.1080/1360786031000101157.

[2] Wiggins RD, Netuveli G, Hyde M, Higgs P, Blane D. The Evaluation of a Self-enumerated Scale of Quality of Life (CASP-19) in the Context of Research on Ageing: A Combination of Exploratory and Confirmatory Approaches. *Social Indicators Research*. 2007;89(1):61-77. doi: 10.1007/s11205-007-9220-5.

[3] Steptoe A, Demakakos P, de Oliveira C, Wardle J. Distinctive biological correlates of positive psychological well-being in older men and women. *Psychosom Med*. 2012 Jun;74(5):501-8. doi: 10.1097/PSY.0b013e31824f82c8.

[4] Gale CR, Cooper C, Deary IJ, Aihie Sayer A. Psychological well-being and incident frailty in men and women: the English Longitudinal Study of Ageing. *Psychological Medicine*. 2014;44(4):697-706. doi: 10.1017/S0033291713001384.

[5] Brooks R. EuroQol: the current state of play. *Health Policy*. 1996;37(1):53-72. doi: 10.1016/0168-8510(96)00822-6.

[6] The EuroQol Group. EuroQol - a new facility for the measurement of health-related quality of life. *Health Policy*. 1990;16(3):199-208. doi: 10.1016/0168-8510(90)90421-9.

[7] Rabin R, Charro Fd. EQ-5D: a measure of health status from the EuroQol Group. *Annals of Medicine*. 2009;33(5):337-43. doi: 10.3109/07853890109002087.

[8] da Rocha NS, Power MJ, Bushnell DM, Fleck MP. The EUROHIS-QOL 8-item index: Comparative psychometric properties to its parent WHOQOL-BREF. *Value in Health*. 2012;15(3):449-57. doi: 10.1016/j.jval.2011.11.035.

[9] Power M. Development of a common instrument for quality of life In: Nosikov A, Gudex C, editors. *EUROHIS: Developing Common Instruments for Health Surveys*. Amsterdam: IOS Press; 2003. p. 145-59.

[10] Schmidt S, Mühlan H, Power M. The EUROHIS-QOL 8-item index: Psychometric results of a cross-cultural field study. *European Journal of Public Health*. 2006;16(4):420-8. doi: 10.1093/eurpub/cki155.

[11] Pinto JM, Fontaine AM, Neri AL. The influence of physical and mental health on life satisfaction is mediated by self-rated health: A study with Brazilian elderly. *Archives of Gerontology and Geriatrics*. 2016;65:104-10. doi: 10.1016/j.archger.2016.03.009.

[12] Yang F, Gu D, Mitnitski A. Frailty and life satisfaction in Shanghai older adults: The roles of age and social vulnerability. *Archives of Gerontology & Geriatrics*. 2016;67:68-73. doi: 10.1016/j.archger.2016.07.001.

[13] St John PD, Tyas SL, Montgomery PR. Life satisfaction and frailty in community-based older adults: Cross-sectional and prospective analyses. *International Psychogeriatrics*. 2013 Oct;25(10):1709-16. doi: 10.1017/S1041610213000902.

[14] Andrews FM, Crandall R. The validity of self-reported well-being. *Social Indicators Research*. 1976;3(1):1-19. doi: 10.1007/BF00286161.

[15] Michalos AC. Satisfaction and happiness. *Social Indicators Research*. 1980;8(4):385–422. doi: 10.1007/BF00461152.

[16] Bowling A. The Psychometric Properties of the Older People's Quality of Life Questionnaire, Compared with the CASP-19 and the WHOQOL-OLD. *Current Gerontology and Geriatrics Research*. 2009;2009:Article 298950. doi: 10.1155/2009/298950.

[17] Bowling A, Stenner P. Which measure of quality of life performs best in older age? A comparison of the OPQOL, CASP-19 and WHOQOL-OLD. *Journal of Epidemiology and Community Health*. 2011;65(3):273-80. doi: 10.1136/jech.2009.087668.

[18] Ament BHL, de Vugt ME, Verhey FRJ, Kempen GIJM. Are physically frail older persons more at risk of adverse outcomes if they also suffer from cognitive, social, and psychological frailty? *European Journal of Ageing*. 2014 Sep;11(3):213-9. doi: 10.1007/s10433-014-0308-x.

[19] Clarke PJ, Marshall VW, Ryff CD, Wheaton B. Measuring Psychological Well-Being in the Canadian Study of Health and Aging. *International Psychogeriatrics*. 2001;13(S1):79-90. doi: 10.1017/s1041610202008013.
[20] Ware JE, Kosinski MA, Keller SD. *SF-12: How to Score the SF-12 Physical and Mental Health Summary Scales*. 2nd ed. Boston, MA: The Health Institute, New England Medical Center; 1995.
[21] Ware JE, Kosinski M, Keller SD. A 12-Item Short-Form Health Survey: construction of scales and preliminary tests of reliability and validity. *Medical care*. 1996;34(3):220-33.
[22] Ware JE. *SF-36 Health Survey: Manual and Interpretation Guide*. 1993.
[23] Ware JE, Sherbourne CD. The MOS 36-item short-form health survey (SF-36): I. Conceptual framework and item selection. *Medical Care*. 1992 Jun;30(6):473-83.
[24] McHorney CA, Ware JE, Raczek AE. The MOS 36-Item Short-Form Health Survey (SF-36): II. Psychometric and clinical tests of validity in measuring physical and mental health constructs. *Medical Care*. 1993;31(3):247-63.
[25] Ware JE, Kosinski M, Bayliss MS, McHorney CA, Rogers WH, Raczek AE. Comparison of methods for the scoring and statistical analysis of SF-36 health profile and summary measures: Summary of results from the Medical Outcomes Study. *Medical Care*. 1995;33(4 Suppl.):AS264-AS79.
[26] Diener E, Emmons RA, Larsen RJ, Griffin S. The Satisfaction With Life Scale. *Journal of Personality Assessment*. 1985 Feb;49(1):71-5. doi: 10.1207/s15327752jpa4901_13.
[27] Pavot W, Diener E, Colvin CR, Sandvik E. Further Validation of the Satisfaction With Life Scale: Evidence for the Cross-Method Convergence of Well-Being Measures. *Journal of Personality Assessment*. 1991 Aug;57(1):149-61. doi: 10.1207/s15327752jpa5701_17.
[28] The WHOQOL Group. Development of the World Health Organization WHOQOL-BREF Quality of Life Assessment. *Psychological Medicine*. 1998;28(3):551-8. doi: 10.1017/S0033291798006667.
[29] Skevington SM, Lotfy M, O'Connell KA. The World Health Organization's WHOQOL-BREF quality of life assessment: psychometric properties and results of the international field trial. A report from the WHOQOL group. *Quality of Life Research*. 2004;13(2):299-310. doi: 10.1023/B:QURE.0000018486.91360.00.
[30] Power M, Quinn K, Schmidt S, WHOQOL-OLD Group. Development of the WHOQOL-OLD module. *Quality of Life Research*. 2005;14(10):2197-214. Power M Quinn K Schmidt S WHOQOL-OLD Group.
