# Supplementary material for: Quality of life is substantially worse for community-dwelling older people living with frailty: systematic review and meta-analysis
Source: Qual Life Res. 2019 Mar 14;28(8):2041–56. doi: 10.1007/s11136-019-02149-1 (PMC6620381; doi:10.1007/s11136-019-02149-1)
Supplement: Supplementary file 5 — Supplementary material 5 (PDF 839 KB) [file 11136_2019_2149_MOESM5_ESM.pdf]

Online Resource 5 for "Quality of life is substantially worse for community-dwelling older people living with frailty: systematic review and meta-analysis". *Quality of Life Research*. Crocker TF*, Brown L, Clegg A, Farley K, Franklin M, Simpkins S, Young J. * Bradford Teaching Hospitals NHS Foundation Trust; tom.crocker@bthft.nhs.uk

Pooled standardised mean differences for QOL measures for people with and without frailty

	Not frail			Frail				Mean difference
Measure / study	Mean	SD	N	Mean	SD	N	Weight	IV, random, 95% C
SF-36 Physical function								
Chang 2012	79.5	19.9	352	54.8	26.2	22	16.6%	1.21 [0.77, 1.65]
Jurschik 2012	68.4	26.3	473	24.3	24.4	50	19.8%	1.69 [1.38, 1.99]
Lenardt 2014	77.5	23.4	164	61.1	27.9	39	18.7%	0.67 [0.32, 1.03]
Lin 2011	83.1	26.5	841	62.7	18.8	92	21.9%	0.79 [0.57, 1.01]
Masel 2009	50.9	31	811	23.3	24.2	200	23.0%	0.93 [0.77, 1.09]
Subtotal (95% CI)			2641			403	100.0%	1.05 [0.73, 1.36]
Heterogeneity: Tau² = 0.11; Chi² = 27.39, df = 4 (P < 0.0001); I² = 85%								
Test for overall effect: Z = 6.51 (P < 0.00001)								
SF-36 Role—Physical								
Chang 2012	76.7	39.5	352	45.5	48.6	22	16.9%	0.78 [0.34, 1.21]
Jurschik 2012	82.8	32.5	473	42.4	46.3	50	19.9%	1.19 [0.88, 1.49]
Lenardt 2014	85.2	32.4	164	71.1	41.1	39	18.8%	0.41 [0.06, 0.76]
Lin 2011	88.7	53.5	841	78.2	38	92	21.7%	0.20 [-0.01, 0.42]
Masel 2009	62.7	45.8	811	31.4	43.2	200	22.7%	0.69 [0.53, 0.85]
Subtotal (95% CI)			2641			403	100.0%	0.64 [0.32, 0.97]
Heterogeneity: Tau² = 0.12; Chi² = 30.75, df = 4 (P < 0.00001); I² = 87%								
Test for overall effect: Z = 3.86 (P = 0.0001)								
SF-36 Bodily pain								
Chang 2012	78.2	21.3	352	59.2	17.4	22	16.1%	0.90 [0.46, 1.33]
Jurschik 2012	40.9	15.8	473	42	19.7	50	20.1%	-0.07 [-0.36, 0.22]
Lenardt 2014	67.4	30.7	164	60.4	30.7	39	18.5%	0.23 [-0.12, 0.58]
Lin 2011	82.4	26.7	841	74.3	19.1	92	22.0%	0.31 [0.09, 0.53]
Masel 2009	68.4	28.7	811	49.7	31	200	23.3%	0.64 [0.48, 0.80]
Subtotal (95% CI)			2641			403	100.0%	0.39 [0.10, 0.69]
Heterogeneity: Tau² = 0.09; Chi² = 24.97, df = 4 (P < 0.0001); I² = 84%								
Test for overall effect: Z = 2.60 (P = 0.009)								
SF-36 General health								
Chang 2012	60.2	14.1	352	48.7	18.9	22	18.2%	0.80 [0.36, 1.23]
Jurschik 2012	68.4	26.3	473	24.3	24.4	50	19.9%	1.69 [1.38, 1.99]
Lenardt 2014	73.9	22.6	164	71.4	17	39	19.4%	0.12 [-0.23, 0.46]
Lin 2011	63.8	31.5	841	49.2	22.3	92	21.0%	0.47 [0.26, 0.69]
Masel 2009	61.2	20.1	811	43.5	20.9	200	21.5%	0.87 [0.71, 1.03]
Subtotal (95% CI)			2641			403	100.0%	0.79 [0.35, 1.23]
Heterogeneity: Tau² = 0.23; Chi² = 55.80, df = 4 (P < 0.00001); I² = 93%								
Test for overall effect: Z = 3.51 (P = 0.0005)								

SF-36 Vitality								
Chang 2012	71.2	17.9	352	55.9	19.4	22	16.4%	0.85 [0.41, 1.28]
Jurschik 2012	58	17.6	473	40.8	18.5	50	20.0%	0.97 [0.67, 1.27]
Lenardt 2014	78.9	21.4	164	75	24.4	39	18.6%	0.18 [-0.17, 0.53]
Lin 2011	74.9	30.2	841	63.2	21.5	92	21.9%	0.40 [0.18, 0.61]
Masel 2009	64.6	21.7	811	44.6	22.6	200	23.1%	0.91 [0.75, 1.07]
Subtotal (95% CI)			2641			403	100.0%	0.66 [0.36, 0.97]
Heterogeneity: $Tau^2 = 0.10$; $Chi^2 = 26.58$, df = 4 ($P < 0.0001$); $I^2 = 85\%$								
Test for overall effect: Z = 4.25 ($P < 0.0001$)								
SF-36 Social functioning								
Chang 2012	89	14.1	352	67.6	21.7	22	17.4%	1.46 [1.02, 1.90]
Jurschik 2012	87.5	21.3	473	59.6	23.9	50	20.0%	1.29 [0.99, 1.59]
Lenardt 2014	89	25	164	85.6	25.6	39	19.2%	0.13 [-0.21, 0.48]
Lin 2011	93.8	24.2	841	80.2	17.2	92	21.3%	0.58 [0.36, 0.79]
Masel 2009	77.1	28.8	811	47.8	33.8	200	22.0%	0.98 [0.82, 1.14]
Subtotal (95% CI)			2641			403	100.0%	0.88 [0.50, 1.25]
Heterogeneity: $Tau^2 = 0.16$; $Chi^2 = 40.21$, df = 4 ($P < 0.00001$); $I^2 = 90\%$								
Test for overall effect: Z = 4.57 ($P < 0.00001$)								
SF-36 Role—Emotional								
Chang 2012	89.1	28.7	352	71.2	44	22	17.4%	0.60 [0.17, 1.03]
Jurschik 2012	87.7	29.8	473	54.4	48.4	50	20.0%	1.04 [0.74, 1.34]
Lenardt 2014	88.4	30.9	164	81.1	36.5	39	19.0%	0.23 [-0.12, 0.58]
Lin 2011	90.9	44.2	841	85.9	31.5	92	21.4%	0.12 [-0.10, 0.33]
Masel 2009	83.5	35.9	811	52.8	48.3	200	22.2%	0.79 [0.63, 0.95]
Subtotal (95% CI)			2641			403	100.0%	0.56 [0.19, 0.92]
Heterogeneity: $Tau^2 = 0.15$; $Chi^2 = 37.79$, df = 4 ($P < 0.00001$); $I^2 = 89\%$								
Test for overall effect: Z = 3.01 ($P = 0.003$)								
SF-36 Mental health								
Chang 2012	79.3	14.4	352	67.5	17.3	22	15.5%	0.81 [0.37, 1.24]
Jurschik 2012	73.9	22.2	473	54.6	29.7	50	19.9%	0.84 [0.54, 1.13]
Lenardt 2014	80.1	21.7	164	76.4	23.4	39	18.1%	0.17 [-0.18, 0.52]
Lin 2011	81	26.5	841	73.7	18.9	92	22.4%	0.28 [0.07, 0.50]
Masel 2009	80.2	18.5	811	66.2	21.4	200	24.1%	0.73 [0.57, 0.89]
Subtotal (95% CI)			2641			403	100.0%	0.56 [0.29, 0.83]
Heterogeneity: $Tau^2 = 0.07$; $Chi^2 = 20.21$, df = 4 ($P = 0.0005$); $I^2 = 80\%$								
Test for overall effect: Z = 4.13 ($P < 0.0001$)								

SF-36 Physical Component Summary								
Chang 2012	48.5	8.3	352	39.5	7.8	22	14.8%	1.09 [0.65, 1.52]
Lin 2011	49.3	10.6	841	42.6	7.6	92	37.1%	0.65 [0.43, 0.86]
Masel 2009	38.8	12	811	29.1	9.9	200	48.1%	0.83 [0.68, 0.99]
Subtotal (95% CI)			2004			314	100.0%	0.80 [0.61, 0.99]
Heterogeneity: $Tau^2 = 0.01$; $Chi^2 = 3.71$, df = 2 (P = 0.16); $I^2 = 46\%$								
Test for overall effect: Z = 8.33 (P < 0.00001)								
SF-36 Mental Component Summary								
Chang 2012	53.6	8.7	352	43.3	12.3	22	29.1%	1.15 [0.71, 1.59]
Lin 2011	55.4	12.4	841	52.6	8.8	92	34.9%	0.23 [0.02, 0.45]
Masel 2009	55.8	9.7	811	46.9	12.7	200	36.0%	0.86 [0.70, 1.02]
Subtotal (95% CI)			2004			314	100.0%	0.72 [0.22, 1.23]
Heterogeneity: $Tau^2 = 0.18$; $Chi^2 = 25.88$, df = 2 (P < 0.00001); $I^2 = 92\%$								
Test for overall effect: Z = 2.82 (P = 0.005)								
WHOQOL-BREF Physical								
Chang 2016	60.9	13.3	161	43.5	13.5	78	33.0%	1.30 [1.00, 1.59]
Gobbens 2013	79.3	11.4	745	60.1	14.7	286	47.5%	1.55 [1.40, 1.70]
Kanauchi 2008	66.3	13.2	77	51.8	14.7	24	19.5%	1.06 [0.58, 1.54]
Subtotal (95% CI)			983			388	100.0%	1.37 [1.11, 1.63]
Heterogeneity: $Tau^2 = 0.03$; $Chi^2 = 5.06$, df = 2 (P = 0.08); $I^2 = 60\%$								
Test for overall effect: Z = 10.20 (P < 0.00001)								
WHOQOL-BREF Psychological								
Chang 2016	65.8	10.1	161	48.8	13	78	33.8%	1.52 [1.22, 1.83]
Gobbens 2013	69.1	10.8	745	58.3	11.6	286	40.4%	0.98 [0.84, 1.12]
Kanauchi 2008	65.2	13.4	77	52.7	14.5	24	25.9%	0.91 [0.43, 1.38]
Subtotal (95% CI)			983			388	100.0%	1.14 [0.76, 1.53]
Heterogeneity: $Tau^2 = 0.09$; $Chi^2 = 10.58$, df = 2 (P = 0.005); $I^2 = 81\%$								
Test for overall effect: Z = 5.83 (P < 0.00001)								
WHOQOL-BREF Social								
Chang 2016	63.8	10.3	161	55.8	10.5	78	33.6%	0.77 [0.49, 1.05]
Gobbens 2013	69.3	14.3	745	56.9	16.1	286	45.2%	0.84 [0.70, 0.98]
Kanauchi 2008	57.3	15.8	77	53.6	17	24	21.2%	0.23 [-0.23, 0.69]
Subtotal (95% CI)			983			388	100.0%	0.68 [0.41, 0.96]
Heterogeneity: $Tau^2 = 0.04$; $Chi^2 = 6.16$, df = 2 (P = 0.05); $I^2 = 68\%$								
Test for overall effect: Z = 4.85 (P < 0.00001)								

WHOQOL-BREF Environment								
Chang 2016	65.6	7.9	161	58	9.5	78	18.8%	0.90 [0.61, 1.18]
Gobbens 2013	76.4	11.1	745	66.1	12.8	286	74.8%	0.89 [0.75, 1.03]
Kanauchi 2008	64.8	9	77	52.1	14.6	24	6.3%	1.19 [0.70, 1.68]
Subtotal (95% CI)			983			388	100.0%	0.91 [0.79, 1.03]
Heterogeneity: Tau² = 0.00; Chi² = 1.39, df = 2 (P = 0.50); I² = 0%								
Test for overall effect: Z = 14.53 (P < 0.00001)								
CASP-19 Total								
ELSA (Hubbard 2014)	44.6	8.4	2778	33.6	9.5	428	56.0%	1.29 [1.18, 1.39]
Wu 2013	38.3	7	682	34.1	8.9	17	44.0%	0.60 [0.11, 1.08]
Subtotal (95% CI)			3460			445	100.0%	0.98 [0.31, 1.65]
Heterogeneity: Tau² = 0.21; Chi² = 7.51, df = 1 (P = 0.006); I² = 87%								
Test for overall effect: Z = 2.87 (P = 0.004)								
CASP-19 Control								
Wu 2013	6.7	1.5	682	7.1	2.6	17	100.0%	-0.26 [-0.74, 0.22]
Subtotal (95% CI)			682			17	100.0%	-0.26 [-0.74, 0.22]
Heterogeneity: Not applicable								
Test for overall effect: Z = 1.06 (P = 0.29)								
CASP-19 Autonomy								
Wu 2013	10.7	1.9	682	9.1	2.8	17	100.0%	0.83 [0.35, 1.31]
Subtotal (95% CI)			682			17	100.0%	0.83 [0.35, 1.31]
Heterogeneity: Not applicable								
Test for overall effect: Z = 3.37 (P = 0.0008)								
CASP-19 Self-realisation								
Wu 2013	11.8	2.8	682	10.5	3.4	17	100.0%	0.46 [-0.02, 0.94]
Subtotal (95% CI)			682			17	100.0%	0.46 [-0.02, 0.94]
Heterogeneity: Not applicable								
Test for overall effect: Z = 1.88 (P = 0.06)								
CASP-19 C+A+S								
ELSA (Gale 2014)	34.1	6.7	2244	28.6	6.22	313	100.0%	0.83 [0.71, 0.95]
Subtotal (95% CI)			2244			313	100.0%	0.83 [0.71, 0.95]
Heterogeneity: Not applicable								
Test for overall effect: Z = 13.47 (P < 0.00001)								
CASP-19 Pleasure								
ELSA (Gale 2014)	10.4	1.7	2244	9.3	1.8	313	94.2%	0.64 [0.52, 0.76]
Wu 2013	8.9	2.8	682	7.4	2.5	17	5.8%	0.54 [0.05, 1.02]
Subtotal (95% CI)			2926			330	100.0%	0.64 [0.52, 0.75]
Heterogeneity: Tau² = 0.00; Chi² = 0.17, df = 1 (P = 0.68); I² = 0%								
Test for overall effect: Z = 10.74 (P < 0.00001)								

OPQOL Total								
Bilotta 2010	120.2	14.5	161	107.4	12.6	78	100.0%	0.92 [0.63, 1.20]
Subtotal (95% CI)			161			78	100.0%	0.92 [0.63, 1.20]
Heterogeneity: Not applicable								
Test for overall effect: Z = 6.36 (P < 0.00001)								
OPQOL Life overall								
Bilotta 2010	13.8	2.8	161	12	3.2	78	100.0%	0.61 [0.34, 0.89]
Subtotal (95% CI)			161			78	100.0%	0.61 [0.34, 0.89]
Heterogeneity: Not applicable								
Test for overall effect: Z = 4.34 (P < 0.0001)								
OPQOL Health								
Bilotta 2010	11.6	3	161	8.2	2.8	78	100.0%	1.15 [0.86, 1.44]
Subtotal (95% CI)			161			78	100.0%	1.15 [0.86, 1.44]
Heterogeneity: Not applicable								
Test for overall effect: Z = 7.81 (P < 0.00001)								
OPQOL Social								
Bilotta 2010	17.5	3.3	161	17.2	3.5	78	100.0%	0.09 [-0.18, 0.36]
Subtotal (95% CI)			161			78	100.0%	0.09 [-0.18, 0.36]
Heterogeneity: Not applicable								
Test for overall effect: Z = 0.64 (P = 0.52)								
OPQOL Independence								
Bilotta 2010	13.2	3.1	161	10.7	2.8	78	100.0%	0.83 [0.55, 1.11]
Subtotal (95% CI)			161			78	100.0%	0.83 [0.55, 1.11]
Heterogeneity: Not applicable								
Test for overall effect: Z = 5.79 (P < 0.00001)								
OPQOL Home								
Bilotta 2010	16.3	2.3	161	15.3	1.8	78	100.0%	0.46 [0.19, 0.74]
Subtotal (95% CI)			161			78	100.0%	0.46 [0.19, 0.74]
Heterogeneity: Not applicable								
Test for overall effect: Z = 3.32 (P = 0.0009)								
OPQOL Psychological								
Bilotta 2010	14.3	2.8	161	12.6	2.6	78	100.0%	0.62 [0.34, 0.90]
Subtotal (95% CI)			161			78	100.0%	0.62 [0.34, 0.90]
Heterogeneity: Not applicable								
Test for overall effect: Z = 4.40 (P < 0.0001)								

OPQOL Finances

Bilotta 2010	13.3	3.1	161	12.8	3.3	78	100.0%	0.16 [-0.11, 0.43]
Subtotal (95% CI)			161			78	100.0%	0.16 [-0.11, 0.43]

Heterogeneity: Not applicable

Test for overall effect: Z = 1.14 (P = 0.25)

OPQOL Activities

Bilotta 2010	20.2	3.3	161	18.7	2.5	78	100.0%	0.49 [0.21, 0.76]
Subtotal (95% CI)			161			78	100.0%	0.49 [0.21, 0.76]

Heterogeneity: Not applicable

Test for overall effect: Z = 3.49 (P = 0.0005)

EQ-VAS

Lahousse 2014	80	11	2670	70	15	163	100.0%	0.89 [0.73, 1.05]
Subtotal (95% CI)			2670			163	100.0%	0.89 [0.73, 1.05]

Heterogeneity: Not applicable

Test for overall effect: Z = 10.88 (P < 0.00001)

SWLS

Simone 2013	27.9	4.7	51	22.8	6.3	44	100.0%	0.92 [0.50, 1.34]
Subtotal (95% CI)			51			44	100.0%	0.92 [0.50, 1.34]

Heterogeneity: Not applicable

Test for overall effect: Z = 4.24 (P < 0.0001)

Test for subgroup differences: $Chi^2 = 133.04$, df = 30 (P < 0.00001), $I^2 = 77.5\%$
