# Supplementary material for: Quality of life is substantially worse for community-dwelling older people living with frailty: systematic review and meta-analysis
Source: Qual Life Res. 2019 Mar 14;28(8):2041–56. doi: 10.1007/s11136-019-02149-1 (PMC6620381; doi:10.1007/s11136-019-02149-1)

Online Resource 6 for “Quality of life is substantially worse for community-dwelling older people living with frailty: systematic review and meta-analysis”. *Quality of Life Research*. Crocker TF*, Brown L, Clegg A, Farley K, Franklin M, Simpkins S, Young J. * Bradford Teaching Hospitals NHS Foundation Trust; tom.crocker@bthft.nhs.uk

Pooled mean differences for QOL measures for people with and without frailty

Measure / study	Not frail			Frail			Weight	Mean difference
	Mean	SD	N	Mean	SD	N		IV, random, 95% CI
SF-36 Physical function								
Chang 2012	79.5	19.9	352	54.8	26.2	22	16.7%	24.70 [13.56, 35.84]
Jurschik 2012	68.4	26.3	473	24.3	24.4	50	20.2%	44.10 [36.93, 51.27]
Lenardt 2014	77.5	23.4	164	61.1	27.9	39	18.2%	16.40 [6.94, 25.86]
Lin 2011	83.1	26.5	841	62.7	18.8	92	22.4%	20.40 [16.16, 24.64]
Masel 2009	50.9	31	811	23.3	24.2	200	22.5%	27.60 [23.63, 31.57]
Subtotal (95% CI)			2641			403	100.0%	26.80 [18.47, 35.13]
Heterogeneity: Tau² = 76.02; Chi² = 35.83, df = 4 (P < 0.00001); I² = 89%								
Test for overall effect: Z = 6.31 (P < 0.00001)								
SF-36 Role—Physical								
Chang 2012	76.7	39.5	352	45.5	48.6	22	14.5%	31.20 [10.48, 51.92]
Jurschik 2012	82.8	32.5	473	42.4	46.3	50	19.6%	40.40 [27.24, 53.56]
Lenardt 2014	85.2	32.2	164	71.1	41.1	39	19.2%	14.10 [0.29, 27.91]
Lin 2011	88.7	53.5	841	78.2	38	92	22.8%	10.50 [1.93, 19.07]
Masel 2009	62.7	45.8	811	31.4	43.2	200	23.9%	31.30 [24.53, 38.07]
Subtotal (95% CI)			2641			403	100.0%	25.03 [13.19, 36.87]
Heterogeneity: Tau² = 140.75; Chi² = 22.32, df = 4 (P = 0.0002); I² = 82%								
Test for overall effect: Z = 4.14 (P < 0.0001)								
SF-36 Bodily pain								
Chang 2012	78.2	21.3	352	59.2	17.4	22	19.2%	19.00 [11.40, 26.60]
Jurschik 2012	40.9	15.8	473	42	19.7	50	20.9%	-1.10 [-6.74, 4.54]
Lenardt 2014	67.4	30.7	164	60.4	30.7	39	16.3%	7.00 [-3.72, 17.72]
Lin 2011	82.4	26.7	841	74.3	19.1	92	21.9%	8.10 [3.80, 12.40]
Masel 2009	68.4	28.7	811	49.7	31	200	21.6%	18.70 [13.97, 23.43]
Subtotal (95% CI)			2641			403	100.0%	10.38 [2.56, 18.20]
Heterogeneity: Tau² = 67.77; Chi² = 34.19, df = 4 (P < 0.00001); I² = 88%								
Test for overall effect: Z = 2.60 (P = 0.009)								
SF-36 General health								
Chang 2012	60.2	14.1	352	48.7	18.9	22	19.1%	11.50 [3.47, 19.53]
Jurschik 2012	68.4	26.3	473	24.3	24.4	50	19.5%	44.10 [36.93, 51.27]
Lenardt 2014	73.9	22.6	164	71.4	17	39	19.9%	2.50 [-3.86, 8.86]

Lin 2011	63.8	31.5	841	49.2	22.3	92	20.4%	14.60 [9.57, 19.63]
Masel 2009	61.2	20.1	811	43.5	20.9	200	21.0%	17.70 [14.49, 20.91]
Subtotal (95% CI)			2641			403	100.0%	18.01 [7.17, 28.86]
Heterogeneity: Tau² = 143.11; Chi² = 77.70, df = 4 (P < 0.00001); I² = 95%								
Test for overall effect: Z = 3.26 (P = 0.001)								
SF-36 Vitality								
Chang 2012	71.2	17.9	352	55.9	19.4	22	15.9%	15.30 [6.98, 23.62]
Jurschik 2012	58	17.6	473	40.8	18.5	50	21.2%	17.20 [11.83, 22.57]
Lenardt 2014	78.9	21.4	164	75	24.4	39	15.9%	3.90 [-4.43, 12.23]
Lin 2011	74.9	30.2	841	63.2	21.5	92	22.2%	11.70 [6.86, 16.54]
Masel 2009	64.6	21.7	811	44.6	22.6	200	24.7%	20.00 [16.53, 23.47]
Subtotal (95% CI)			2641			403	100.0%	14.25 [9.17, 19.32]
Heterogeneity: Tau² = 24.09; Chi² = 16.46, df = 4 (P = 0.002); I² = 76%								
Test for overall effect: Z = 5.50 (P < 0.00001)								
SF-36 Social functioning								
Chang 2012	89	14.1	352	67.6	21.7	22	17.7%	21.40 [12.21, 30.59]
Jurschik 2012	87.5	21.3	473	59.6	3.9	50	22.1%	27.90 [25.70, 30.10]
Lenardt 2014	89	25	164	85.6	25.6	39	18.0%	3.40 [-5.50, 12.30]
Lin 2011	93.8	24.2	841	80.2	17.2	92	21.4%	13.60 [9.72, 17.48]
Masel 2009	77.1	28.8	811	47.8	33.8	200	20.8%	29.30 [24.21, 34.39]
Subtotal (95% CI)			2641			403	100.0%	19.57 [11.12, 28.02]
Heterogeneity: Tau² = 82.91; Chi² = 64.74, df = 4 (P < 0.00001); I² = 94%								
Test for overall effect: Z = 4.54 (P < 0.00001)								
SF-36 Role—Emotional								
Chang 2012	89.1	28.7	352	71.2	44	22	16.3%	17.90 [-0.73, 36.53]
Jurschik 2012	87.7	29.8	473	54.4	48.4	50	19.1%	33.30 [19.62, 46.98]
Lenardt 2014	88.4	30.9	164	81.1	36.5	39	19.8%	7.30 [-5.09, 19.69]
Lin 2011	90.9	44.2	841	85.9	31.5	92	22.4%	5.00 [-2.10, 12.10]
Masel 2009	83.5	35.9	811	52.8	48.3	200	22.4%	30.70 [23.56, 37.84]
Subtotal (95% CI)			2641			403	100.0%	18.72 [5.35, 32.09]
Heterogeneity: Tau² = 194.81; Chi² = 32.73, df = 4 (P < 0.00001); I² = 88%								
Test for overall effect: Z = 2.74 (P = 0.006)								
SF-36 Mental health								
Chang 2012	79.3	14.4	352	67.5	17.3	22	17.3%	11.80 [4.42, 19.18]
Jurschik 2012	73.9	22.2	473	54.6	29.7	50	15.1%	19.30 [10.83, 27.77]
Lenardt 2014	80.1	21.7	164	76.4	23.4	39	15.9%	3.70 [-4.36, 11.76]
Lin 2011	81	26.5	841	73.7	18.9	92	24.6%	7.30 [3.04, 11.56]

Masel 2009	80.2	18.5	811	66.2	21.4	200	27.1%	14.00 [10.77, 17.23]
Subtotal (95% CI)			2641			403	100.0%	11.13 [6.56, 15.71]
Heterogeneity: Tau² = 17.39; Chi² = 12.90, df = 4 (P = 0.01); I² = 69%								
Test for overall effect: Z = 4.77 (P < 0.00001)								
SF-36 Physical Component Summary								
Chang 2012	48.5	8.3	352	39.5	7.8	22	22.5%	9.00 [5.63, 12.37]
Lin 2011	49.3	10.6	841	42.6	7.6	92	38.2%	6.70 [4.99, 8.41]
Masel 2009	38.8	12	811	29.1	9.9	200	39.3%	9.70 [8.10, 11.30]
Subtotal (95% CI)			2004			314	100.0%	8.40 [6.24, 10.55]
Heterogeneity: Tau² = 2.40; Chi² = 6.45, df = 2 (P = 0.04); I² = 69%								
Test for overall effect: Z = 7.64 (P < 0.00001)								
SF-36 Mental Component Summary								
Chang 2012	53.6	8.7	352	43.3	12.3	22	26.9%	10.30 [5.08, 15.52]
Lin 2011	55.4	12.4	841	52.6	8.8	92	36.5%	2.80 [0.82, 4.78]
Masel 2009	55.8	9.7	811	46.9	12.7	200	36.7%	8.90 [7.02, 10.78]
Subtotal (95% CI)			2004			314	100.0%	7.05 [2.18, 11.93]
Heterogeneity: Tau² = 15.95; Chi² = 21.54, df = 2 (P < 0.0001); I² = 91%								
Test for overall effect: Z = 2.83 (P = 0.005)								
WHOQOL-BREF Physical								
Chang 2016	60.9	13.3	161	43.5	13.5	78	24.4%	17.40 [13.77, 21.03]
Gobbens 2013	79.3	11.4	745	60.1	14.7	286	67.4%	19.20 [17.31, 21.09]
Kanauchi 2008	66.3	13.2	77	51.8	14.7	24	8.2%	14.50 [7.92, 21.08]
Subtotal (95% CI)			983			388	100.0%	18.38 [16.46, 20.30]
Heterogeneity: Tau² = 0.49; Chi² = 2.30, df = 2 (P = 0.32); I² = 13%								
Test for overall effect: Z = 18.75 (P < 0.00001)								
WHOQOL-BREF Psychological								
Chang 2016	65.8	10.1	161	48.8	13	78	35.5%	17.00 [13.72, 20.28]
Gobbens 2013	69.1	10.8	745	58.3	11.6	286	41.3%	10.80 [9.25, 12.35]
Kanauchi 2008	65.2	13.4	77	52.7	14.5	24	23.1%	12.50 [5.97, 19.03]
Subtotal (95% CI)			983			388	100.0%	13.40 [8.80, 17.99]
Heterogeneity: Tau² = 12.66; Chi² = 11.24, df = 2 (P = 0.004); I² = 82%								
Test for overall effect: Z = 5.72 (P < 0.00001)								
WHOQOL-BREF Social								
Chang 2016	63.8	10.3	161	55.8	10.5	78	39.1%	8.00 [5.18, 10.82]
Gobbens 2013	69.3	14.3	745	56.9	16.1	286	42.2%	12.40 [10.27, 14.53]
Kanauchi 2008	57.3	15.8	77	53.6	17	24	18.7%	3.70 [-3.96, 11.36]
Subtotal (95% CI)			983			38	100.0	9.06 [4.80, 13.31]

						8	%	

Heterogeneity: $Tau^2 = 9.99$; $Chi^2 = 9.10$, df = 2 (P = 0.01); $I^2 = 78\%$

Test for overall effect: Z = 4.17 (P < 0.0001)

WHOQOL-BREF Environment

Chang 2016	65.6	7.9	161	58	9.5	78	38.7%	7.60 [5.16, 10.04]
Gobbens 2013	76.4	11.1	745	66.1	12.8	286	49.4%	10.30 [8.62, 11.98]
Kanauchi 2008	64.8	9	77	52.1	14.6	24	11.9%	12.70 [6.52, 18.88]
Subtotal (95% CI)			983			388	100.0%	9.54 [7.19, 11.89]

Heterogeneity: $Tau^2 = 2.16$; $Chi^2 = 4.22$, df = 2 (P = 0.12); $I^2 = 53\%$

Test for overall effect: Z = 7.96 (P < 0.00001)

CASP-19 Total

ELSA (Hubbard 2014)	44.6	8.4	2778	33.6	9.5	428	54.9%	11.00 [10.05, 11.95]
Wu 2013	38.3	7	682	34.1	8.9	17	45.1%	4.20 [-0.06, 8.46]
Subtotal (95% CI)			3460			445	100.0%	7.93 [1.30, 14.56]

Heterogeneity: $Tau^2 = 20.64$; $Chi^2 = 9.31$, df = 1 (P = 0.002); $I^2 = 89\%$

Test for overall effect: Z = 2.34 (P = 0.02)

Study or Subgroup	Not frail Mean	SD	Total	Frail Mean	SD	Total	Weight	Mean Difference IV, Random, 95% CI
**1.1.1 SF-36 Physical function**								
Chang 2012	79.5	19.9	352	54.8	26.2	22	16.7%	24.70 [13.56, 35.84]
Jurschik 2012	68.4	26.3	473	24.3	24.4	50	20.2%	44.10 [36.93, 51.27]
Lenardt 2014	77.5	23.4	164	61.1	27.9	39	18.2%	16.40 [6.94, 25.86]
Lin 2011	83.1	26.5	841	62.7	18.8	92	22.4%	20.40 [16.16, 24.64]
Masel 2009	50.9	31	811	23.3	24.2	200	22.5%	27.60 [23.63, 31.57]
**Subtotal (95% CI)**			**2641**			**403**	**100.0%**	**26.80 [18.47, 35.13]**
Heterogeneity: Tau² = 76.02; Chi² = 35.83, df = 4 (P < 0.00001); I² = 89%								
Test for overall effect: Z = 6.31 (P < 0.00001)								
**1.1.2 SF-36 Role—Physical**								
Chang 2012	76.7	39.5	352	45.5	48.6	22	14.5%	31.20 [10.48, 51.92]
Jurschik 2012	82.8	32.5	473	42.4	46.3	50	19.6%	40.40 [27.24, 53.56]
Lenardt 2014	85.2	32.2	164	71.1	41.1	39	19.2%	14.10 [0.29, 27.91]
Lin 2011	88.7	53.5	841	78.2	38	92	22.8%	10.50 [1.93, 19.07]
Masel 2009	62.7	45.8	811	31.4	43.2	200	23.9%	31.30 [24.53, 38.07]
**Subtotal (95% CI)**			**2641**			**403**	**100.0%**	**25.03 [13.19, 36.87]**
Heterogeneity: Tau² = 140.75; Chi² = 22.32, df = 4 (P = 0.0002); I² = 82%								
Test for overall effect: Z = 4.14 (P < 0.0001)								
**1.1.3 SF-36 Bodily pain**								
Chang 2012	78.2	21.3	352	59.2	17.4	22	19.2%	19.00 [11.40, 26.60]
Jurschik 2012	40.9	15.8	473	42	19.7	50	20.9%	-1.10 [-6.74, 4.54]
Lenardt 2014	67.4	30.7	164	60.4	30.7	39	16.3%	7.00 [-3.72, 17.72]
Lin 2011	82.4	26.7	841	74.3	19.1	92	21.9%	8.10 [3.80, 12.40]
Masel 2009	68.4	28.7	811	49.7	31	200	21.6%	18.70 [13.97, 23.43]
**Subtotal (95% CI)**			**2641**			**403**	**100.0%**	**10.38 [2.56, 18.20]**
Heterogeneity: Tau² = 67.77; Chi² = 34.19, df = 4 (P < 0.00001); I² = 88%								
Test for overall effect: Z = 2.60 (P = 0.009)								
**1.1.4 SF-36 General health**								
Chang 2012	60.2	14.1	352	48.7	18.9	22	19.1%	11.50 [3.47, 19.53]
Jurschik 2012	68.4	26.3	473	24.3	24.4	50	19.5%	44.10 [36.93, 51.27]
Lenardt 2014	73.9	22.6	164	71.4	17	39	19.9%	2.50 [-3.86, 8.86]
Lin 2011	63.8	31.5	841	49.2	22.3	92	20.4%	14.60 [9.57, 19.63]
Masel 2009	61.2	20.1	811	43.5	20.9	200	21.0%	17.70 [14.49, 20.91]
**Subtotal (95% CI)**			**2641**			**403**	**100.0%**	**18.01 [7.17, 28.86]**
Heterogeneity: Tau² = 143.11; Chi² = 77.70, df = 4 (P < 0.00001); I² = 95%								
Test for overall effect: Z = 3.26 (P = 0.001)								
**1.1.5 SF-36 Vitality**								
Chang 2012	71.2	17.9	352	55.9	19.4	22	15.9%	15.30 [6.98, 23.62]
Jurschik 2012	58	17.6	473	40.8	18.5	50	21.2%	17.20 [11.83, 22.57]
Lenardt 2014	78.9	21.4	164	75	24.4	39	15.9%	3.90 [-4.43, 12.23]
Lin 2011	74.9	30.2	841	63.2	21.5	92	22.2%	11.70 [6.86, 16.54]
Masel 2009	64.6	21.7	811	44.6	22.6	200	24.7%	20.00 [16.53, 23.47]
**Subtotal (95% CI)**			**2641**			**403**	**100.0%**	**14.25 [9.17, 19.32]**
Heterogeneity: Tau² = 24.09; Chi² = 16.46, df = 4 (P = 0.002); I² = 76%								
Test for overall effect: Z = 5.50 (P < 0.00001)								
**1.1.6 SF-36 Social functioning**								
Chang 2012	89	14.1	352	67.6	21.7	22	17.7%	21.40 [12.21, 30.59]
Jurschik 2012	87.5	21.3	473	59.6	3.9	50	22.1%	27.90 [25.70, 30.10]
Lenardt 2014	89	25	164	85.6	25.6	39	18.0%	3.40 [-5.50, 12.30]
Lin 2011	93.8	24.2	841	80.2	17.2	92	21.4%	13.60 [9.72, 17.48]
Masel 2009	77.1	28.8	811	47.8	33.8	200	20.8%	29.30 [24.21, 34.39]
**Subtotal (95% CI)**			**2641**			**403**	**100.0%**	**19.57 [11.12, 28.02]**
Heterogeneity: Tau² = 82.91; Chi² = 64.74, df = 4 (P < 0.00001); I² = 94%								
Test for overall effect: Z = 4.54 (P < 0.00001)								
**1.1.7 SF-36 Role—Emotional**								
Chang 2012	89.1	28.7	352	71.2	44	22	16.3%	17.90 [-0.73, 36.53]
Jurschik 2012	87.7	29.8	473	54.4	48.4	50	19.1%	33.30 [19.62, 46.98]
Lenardt 2014	88.4	30.9	164	81.1	36.5	39	19.8%	7.30 [-5.09, 19.69]
Lin 2011	90.9	44.2	841	85.9	31.5	92	22.4%	5.00 [-2.10, 12.10]
Masel 2009	83.5	35.9	811	52.8	48.3	200	22.4%	30.70 [23.56, 37.84]
**Subtotal (95% CI)**			**2641**			**403**	**100.0%**	**18.72 [5.35, 32.09]**
Heterogeneity: Tau² = 194.81; Chi² = 32.73, df = 4 (P < 0.00001); I² = 88%								
Test for overall effect: Z = 2.74 (P = 0.006)								
**1.1.8 SF-36 Mental health**								
Chang 2012	79.3	14.4	352	67.5	17.3	22	17.3%	11.80 [4.42, 19.18]
Jurschik 2012	73.9	22.2	473	54.6	29.7	50	15.1%	19.30 [10.83, 27.77]
Lenardt 2014	80.1	21.7	164	76.4	23.4	39	15.9%	3.70 [-4.36, 11.76]
Lin 2011	81	26.5	841	73.7	18.9	92	24.6%	7.30 [3.04, 11.56]
Masel 2009	80.2	18.5	811	66.2	21.4	200	27.1%	14.00 [10.77, 17.23]
**Subtotal (95% CI)**			**2641**			**403**	**100.0%**	**11.13 [6.56, 15.71]**
Heterogeneity: Tau² = 17.39; Chi² = 12.90, df = 4 (P = 0.01); I² = 69%								
Test for overall effect: Z = 4.77 (P < 0.00001)								
**1.1.9 SF-36 Physical Component Summary**								
Chang 2012	48.5	8.3	352	39.5	7.8	22	22.5%	9.00 [5.63, 12.37]
Lin 2011	49.3	10.6	841	42.6	7.6	92	38.2%	6.70 [4.99, 8.41]
Masel 2009	38.8	12	811	29.1	9.9	200	39.3%	9.70 [8.10, 11.30]
**Subtotal (95% CI)**			**2004**			**314**	**100.0%**	**8.40 [6.24, 10.55]**
Heterogeneity: Tau² = 2.40; Chi² = 6.45, df = 2 (P = 0.04); I² = 69%								
Test for overall effect: Z = 7.64 (P < 0.00001)								
**1.1.10 SF-36 Mental Component Summary**								
Chang 2012	53.6	8.7	352	43.3	12.3	22	26.9%	10.30 [5.08, 15.52]
Lin 2011	55.4	12.4	841	52.6	8.8	92	36.5%	2.80 [0.82, 4.78]
Masel 2009	55.8	9.7	811	46.9	12.7	200	36.7%	8.90 [7.02, 10.78]
**Subtotal (95% CI)**			**2004**			**314**	**100.0%**	**7.05 [2.18, 11.93]**
Heterogeneity: Tau² = 15.95; Chi² = 21.54, df = 2 (P < 0.0001); I² = 91%								
Test for overall effect: Z = 2.83 (P = 0.005)								

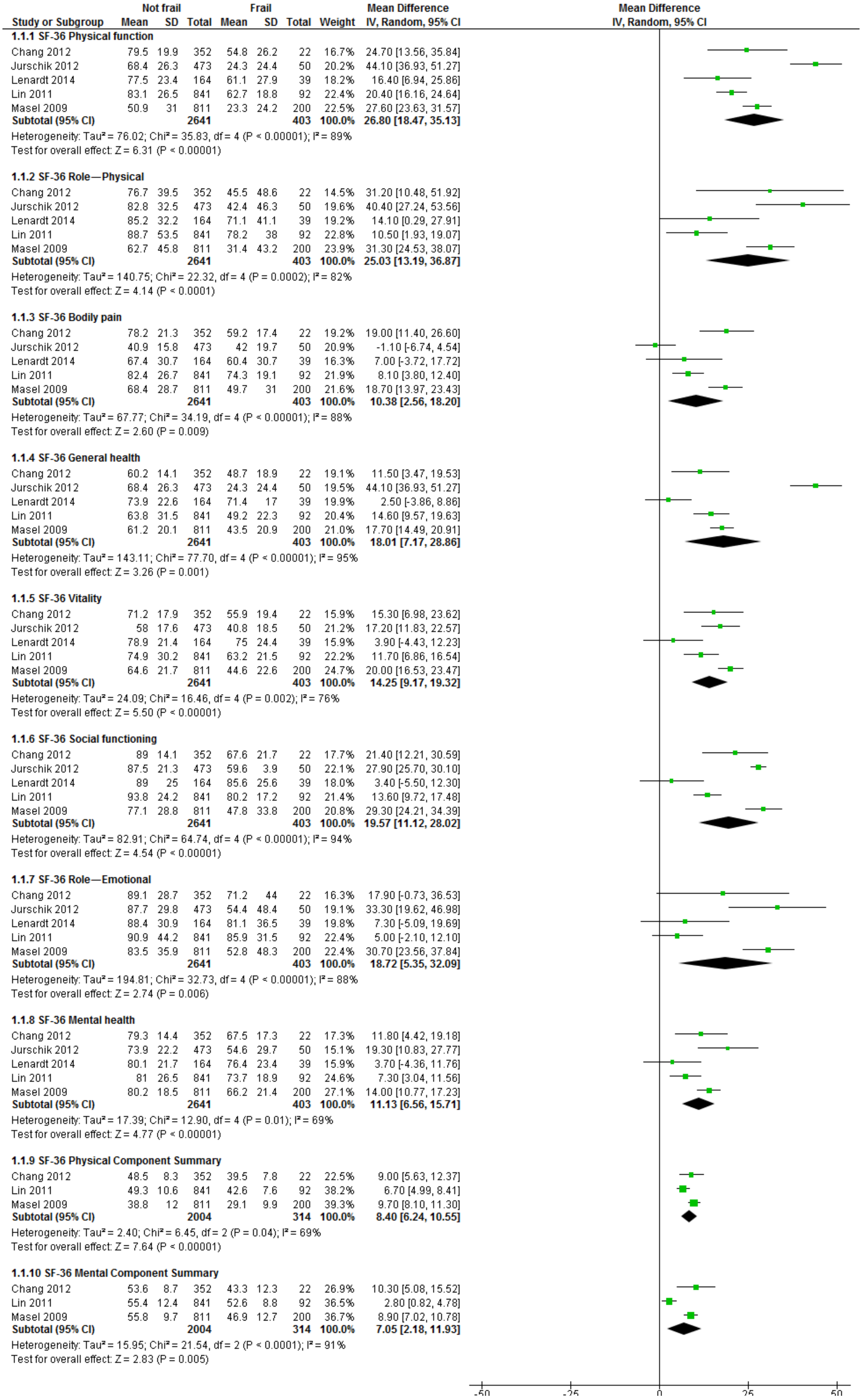

Study or Subgroup	Not frail Mean	Not frail SD	Not frail Total	Frail Mean	Frail SD	Frail Total	Weight	Mean Difference IV, Random, 95% CI
**1.1.11 WHOQOL-BREF Physical**								
Chang 2016	60.9	13.3	161	43.5	13.5	78	24.4%	17.40 [13.77, 21.03]
Gobbens 2013	79.3	11.4	745	60.1	14.7	286	67.4%	19.20 [17.31, 21.09]
Kanauchi 2008	66.3	13.2	77	51.8	14.7	24	8.2%	14.50 [7.92, 21.08]
**Subtotal (95% CI)**			**983**			**388**	**100.0%**	**18.38 [16.46, 20.30]**
Heterogeneity: Tau² = 0.49; Chi² = 2.30, df = 2 (P = 0.32); I² = 13%								
Test for overall effect: Z = 18.75 (P < 0.00001)								
**1.1.12 WHOQOL-BREF Psychological**								
Chang 2016	65.8	10.1	161	48.8	13	78	35.5%	17.00 [13.72, 20.28]
Gobbens 2013	69.1	10.8	745	58.3	11.6	286	41.3%	10.80 [9.25, 12.35]
Kanauchi 2008	65.2	13.4	77	52.7	14.5	24	23.1%	12.50 [5.97, 19.03]
**Subtotal (95% CI)**			**983**			**388**	**100.0%**	**13.40 [8.80, 17.99]**
Heterogeneity: Tau² = 12.66; Chi² = 11.24, df = 2 (P = 0.004); I² = 82%								
Test for overall effect: Z = 5.72 (P < 0.00001)								
**1.1.13 WHOQOL-BREF Social**								
Chang 2016	63.8	10.3	161	55.8	10.5	78	39.1%	8.00 [5.18, 10.82]
Gobbens 2013	69.3	14.3	745	56.9	16.1	286	42.2%	12.40 [10.27, 14.53]
Kanauchi 2008	57.3	15.8	77	53.6	17	24	18.7%	3.70 [-3.96, 11.36]
**Subtotal (95% CI)**			**983**			**388**	**100.0%**	**9.06 [4.80, 13.31]**
Heterogeneity: Tau² = 9.99; Chi² = 9.10, df = 2 (P = 0.01); I² = 78%								
Test for overall effect: Z = 4.17 (P < 0.0001)								
**1.1.14 WHOQOL-BREF Environment**								
Chang 2016	65.6	7.9	161	58	9.5	78	38.7%	7.60 [5.16, 10.04]
Gobbens 2013	76.4	11.1	745	66.1	12.8	286	49.4%	10.30 [8.62, 11.98]
Kanauchi 2008	64.8	9	77	52.1	14.6	24	11.9%	12.70 [6.52, 18.88]
**Subtotal (95% CI)**			**983**			**388**	**100.0%**	**9.54 [7.19, 11.89]**
Heterogeneity: Tau² = 2.16; Chi² = 4.22, df = 2 (P = 0.12); I² = 53%								
Test for overall effect: Z = 7.96 (P < 0.00001)								
**1.1.15 CASP-19 Total**								
ELSA (Hubbard 2014)	44.6	8.4	2778	33.6	9.5	428	54.9%	11.00 [10.05, 11.95]
Wu 2013	38.3	7	682	34.1	8.9	17	45.1%	4.20 [-0.06, 8.46]
**Subtotal (95% CI)**			**3460**			**445**	**100.0%**	**7.93 [1.30, 14.56]**
Heterogeneity: Tau² = 20.64; Chi² = 9.31, df = 1 (P = 0.002); I² = 89%								
Test for overall effect: Z = 2.34 (P = 0.02)								

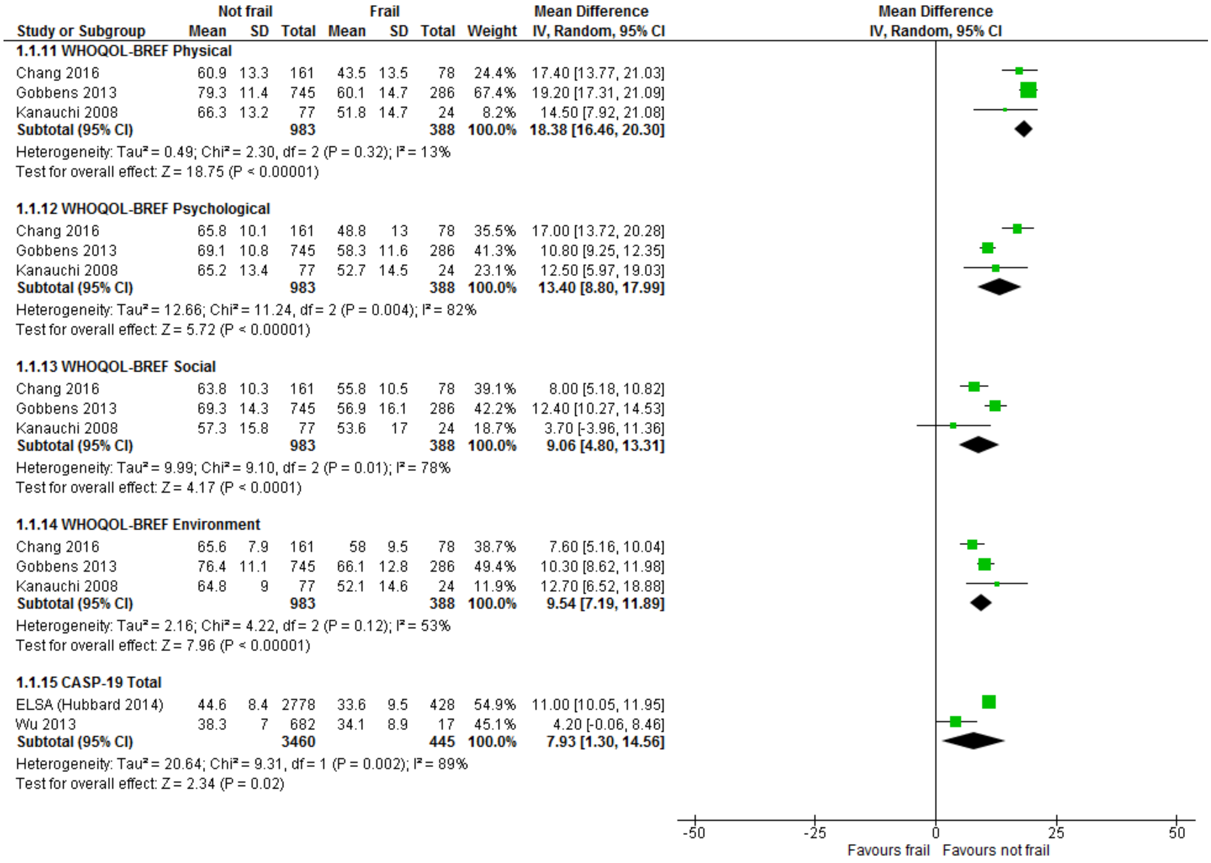

Supplement: Supplementary file 6 — Supplementary material 6 (PDF 636 KB) [file 11136_2019_2149_MOESM6_ESM.pdf]
