# Supplementary material for: Quality of life is substantially worse for community-dwelling older people living with frailty: systematic review and meta-analysis
Source: Qual Life Res. 2019 Mar 14;28(8):2041–56. doi: 10.1007/s11136-019-02149-1 (PMC6620381; doi:10.1007/s11136-019-02149-1)
Supplement: Supplementary file 7 — Supplementary material 7 (PDF 208 KB) [file 11136_2019_2149_MOESM7_ESM.pdf]

Online Resource 7 for “Quality of life is substantially worse for community-dwelling older people living with frailty: systematic review and meta-analysis”. *Quality of Life Research*. Crocker TF*, Brown L, Clegg A, Farley K, Franklin M, Simpkins S, Young J. * Bradford Teaching Hospitals NHS Foundation Trust; tom.crocker@bthft.nhs.uk

# Analyses of associations between QOL and frailty from individual studies

| Study details | Numbers of participants and QOL scores by frailty category [a] | | | Reported analyses of associations between frailty and QOL | Covariates adjusted for |
|---|---|---|---|---|---|
| | Fit [†] | Pre-frail [‡] | Frail [§] | | |
| Ament 2014<br><br>N=334<br>Frailty:<br>Groningen Frailty indicator<br>QoL: Single item general QOL assessment (unreferenced) | n=0 [b] | n=0 [b] | n=334 (100%)<br><br>QoL excellent, very good or good: 50%,<br>QoL moderate or bad: 50% | Analyses only conducted for domains of frailty | Age, sex, baseline quality of life, multimorbidity |
| CSHA<br><br>N=5,703<br>Frailty:<br>Cumulative Deficit Model<br>QoL: Ryff Psychological Well-Being scale | n=4677 (82%)<br><br>QoL scores not reported by frailty category | n=0 [c] | n=1026 (18%) | Baseline Pearson's r = 0.23, P < 0.001<br><br>Linear regression (QOL dependent)<br>Coefficient for frailty (95% CI) p-value:<br>Ryff psychological well-being index<br>Total 0.29 (0.22 to 0.36) ***<br>- Autonomy 0.012 (−0.0069 to 0.031) -<br>- Mastery 0.12 (0.099 to 0.13) ***<br>- Acceptance 0.071 (0.049 to 0.092) ***<br>- Purpose −0.002 (−0.028 to 0.023) -<br>- Relations 0.047 (0.024 to 0.069) ***<br>- Growth 0.057 (0.033 to 0.081) ***<br><br>AUC = 0.59 [95% confidence interval (CI) 0.54 to 0.64], ***<br><br>logistic regression model, (five-year frailty dependent)<br>Baseline Ryff psychological well-being scale did not significantly predict frailty [p = 0.216] (n=557)<br><br>Mediation model while covarying for other pertinent factors, PWB -> Frailty, and via Depression (n = 557):<br>Coefficent (95% CI), p<br>Total -0.186 (-0.347 to -0.025) *<br>Direct -0.108 (-0.278 to 0.062) -<br>Indirect -0.074 (-0.135 to -0.023) **<br>indicating a full mediation relationship. | Regression analysis adjusted for age, gender, education, mental health, and 3MS score.<br><br>Logistic regression and mediation models adjusted for age, gender and baseline frailty. Mediation via depression in mediation model |

| Study details | Numbers of participants and QOL scores by frailty category [a] | | | | Reported analyses of associations between frailty and QOL | Covariates adjusted for |
|---|---|---|---|---|---|---|
| | | Fit † | Pre-frail ‡ | Frail § | | |
| Bilotta 2010 | n=72 (30%) | | n=89 (37%) | n=78 (33%) | | |
| N=239 | OPQOL, mean (SD) | | | | Between group differences: | |
| Frailty: Study of | total | 125.9 (13.2) | 115.6 (13.9) | 107.4 (12.6) | * | |
| Osteoporotic | - life overall | 14.9 (2.4) | 13.0 (2.8) | 12.0 (3.2) | * | |
| Fractures criteria | - health | 12.9 (2.6) | 10.5 (2.8) | 8.2 (2.8) | * | |
| | - social | 17.8 (3.2) | 17.2 (3.3) | 17.2 (3.5) | - | |
| QoL: Older | - independence | 14.2 (2.9) | 12.4 (3.0) | 10.7 (2.8) | * | |
| People's Quality | - home | 16.7 (2.2) | 15.9 (2.4) | 15.3 (1.8) | * | |
| of Life | - psychological | 15.1 (2.5) | 13.7 (2.9) | 12.6 (2.6) | * | |
| questionnaire | - finances | 13.5 (3.2) | 13.1 (3.0) | 12.8 (3.3) | - | |
| | - activities | 20.8 (3.5) | 19.7 (3.1) | 18.7 (2.5) | * | |
| | | | | | Linear regression analysis,<br>OPQOL total as dependent variable,<br>Being frail unstandardized coefficient (95% CI):<br>-6.36 (-10.37 to -2.35) * | age, basic ADLs instrumental ADLs, MMSE, depression, CIRS m, fell in past year, number of drugs |
| Chang 2012 | n=117 (31%) | | n=235 (63%) | n=22 (6%) | | |
| N=374 | SF-36, mean(SD) | | | | ANOVA: | |
| Frailty: Fried | Physical function | 83.3 (18.7) | 77.6 (20.2) | 54.8 (26.16) | *** | |
| QoL: SF-36 | Role physical | 75.9 (40.7) | 77.1 (39.0) | 45.5 (48.6) | ** | |
| | Bodily pain | 80.3 (20.5) | 77.1 (21.6) | 59.2 (17.4) | *** | |
| | General health | 60.3 (11.5) | 60.1 (15.2) | 48.7 (18.9) | ** | |
| | Vitality | 77.9 (16.4) | 67.8 (17.7) | 55.9 (19.4) | *** | |
| | Social functioning | 91.7 (13.4) | 87.7 (14.3) | 67.6 (21.7) | *** | |
| | Role emotional | 92.6 (24.8) | 87.4 (30.3) | 71.2 (44.0) | ** | |
| | Mental health | 84.2 (14.2) | 76.8 (13.9) | 67.5 (17.3) | *** | |
| | PCS | 48.6 (8.2) | 48.4 (8.4) | 39.5 (7.8) | *** | |
| | MCS | 56.8 (7.7) | 52.0 (8.8) | 43.3 (12.3) | *** | |
| | | | | | Multivariate linear regression:-<br>PCS as dependent variable, β (95% CI):<br>Prefrail 1.461 (-0.499 to 3.421)<br>Frail -6.289** (-10.398 to -2.181)<br>$R^2$ = 0.131 | age, number of co-morbidities, living alone, falls in the previous year, arthritis, peptic ulcer disease, and depression |
| | | | | | MCS as dependent variable, β (95% CI):<br>Prefrail -3.772*** (-5.731 to -1.813)<br>Frail -9.436*** (-13.543 to -5.329)<br>$R^2$ = 0.248 | |

| Study details | Numbers of participants and QOL scores by frailty category [a]<br>Fit † | <br>Pre-frail ‡ | <br>Frail § | Reported analyses of associations between frailty and QOL | Covariates adjusted for |
|---|---|---|---|---|---|
| Chang 2016<br><br>N=239<br>Frailty: Study of Osteoporotic Fractures criteria<br><br>QoL: WHOQOL-BREF | n=72 (30%)<br><br>WHOQOL-BREF, mean (SD):<br>Physical 3.65 (0.49)<br>Psychological 3.66 (0.42)<br>Social 3.58 (0.44)<br>Environment 3.64 (0.35) | n=89 (37%)<br><br><br>3.26 (0.50)<br>3.61 (0.39)<br>3.53 (0.39)<br>3.61 (0.29) | n=78 (33%)<br><br><br>2.74 (0.54)<br>2.95 (0.52)<br>3.23 (0.42)<br>3.32 (0.38) | Between group differences (Chi-squared test):<br>p-value; Scheffe<br>***; †>§,‡; ‡>§<br>***; †>§; ‡>§<br>***; †>§; ‡>§<br>***; †>§; ‡>§<br><br>Multiple linear regressions; QOL subscales dependent. Independent binary frailty variable B coefficients (95% CIs):-<br>Physical -0.01 (-0.15 to 0.12)<br>Psychological -0.13 (-0.28 to 0.01)<br>Social -0.12 (-0.28 to 0.03)<br>Environment -0.16 (-0.30 to -0.02)* | Regression adjusted for sex, age, education, employment, has income, lives alone, number of chronic diseases, fell last year, fracture last year, smoked ever, consumed alcohol last month, self-perceived health, self-perceived happiness, BMI, waist circumference, SBP, DBP, BMD, grip strength |
| Coelho 2015<br><br>N=252<br>Frailty: Tilburg<br>QoL: WHOQOL-OLD and EUROHIS-QOL-8 | n=39 (15%)<br><br>QoL scores not reported by frailty category | n=121 (48%) | n=92 (37%) | Correlation coefficient (r), coefficient of determination ($R^2$), p-value:<br>WHOQOL-OLD and TFI, -0.65, 42.1%, ***<br>EUROHIS-QOL-8 and TFI, -0.62, 38.7%, *** | |
| ELSA (Gale 2014)<br><br>N=2,557<br>Frailty: Cumulative Deficit Model<br>QoL: CASP-19 | n=1186 (46%)<br><br>CASP-19, mean (SD):<br>Total 46.0 (7.63)<br>Hedonic [d] 10.6 (1.67)<br>Eudaimonic [e] 35.3 (6.47) | n=1058 (41%)<br><br><br>42.8 (7.96)<br>10.1 (1.66)<br>32.7 (6.79) | n=313 (12%)<br><br><br>38.0 (7.65)<br>9.34 (1.80)<br>28.6 (6.22) | Differences between groups (ANOVA, Chi-squared test): p-value<br>† and ‡; ‡ and §<br>*** ***<br>*** ***<br>*** ***<br><br>Multinomial logistic regressions:-<br>RR (95% CI), for incident (pre-)frailty (model a):-<br>*Pre-frailty* *Frailty*<br>CASP-19 Total 0.69 (0.63 to 0.77)<br>- Hedonic [d] 0.75 (0.68 to 0.83)<br>- Eudaimonic [e] 0.70 (0.63 to 0.78)<br><br>RR (95% CI), for incident (pre-)frailty (model b, fully adjusted):-<br>*Pre-frailty* *Frailty*<br>Total score 0.79 (0.71 to 0.89)<br>- Hedonic [d] 0.83 (0.74 to 0.92)<br>- Eudaimonic [e] 0.76 (0.72 to 0.90)<br><br>Four-year PWB total score (95% CI):<br>*Incidence of* *model a* *model b, fully adjusted*<br>pre-frailty -1.58 (-0.93 to -2.22)<br>frailty -3.70 (-1.99 to -5.41)<br><br>Also significant relative declines in hedonic and eudaimonic scores for incidence of pre-frailty and frailty (data not shown). | All regression models are adjusted for:<br>(a) age, sex and baseline value of the dependent variable (i.e. frailty status or psychological well-being).<br><br>(b) Fully adjusted models additionally include: household wealth, smoking status, number of chronic physical diseases, BMI, depressive symptom score, cognitive function. |

| Study details | Numbers of participants and QOL scores by frailty category [a] | | | Reported analyses of associations between frailty and QOL | Covariates adjusted for |
|---|---|---|---|---|---|
| | Fit † | Pre-frail ‡ | Frail § | | |
| ELSA (Hubbard 2014)<br><br>N=3,206<br>Frailty: Cumulative Deficit Model<br>QoL: CASP-19 | Very fit: n=1879 (59%); Well: n =343 (11%)<br><br>CASP-19 total, mean {95% CI}<br>Very fit: 46.4 {46.0 to 46.7}; Well: 42.3 {41.6 to 43.1} | Vulnerable: n=556 (17%)<br><br>39.8 {39.1 to 40.4} | Frail: n=428 (13%)<br><br>33.6 {32.7 to 34.5} | Pearson correlation coefficient (r):<br>CASP-19: -0.58<br><br>Linear regressions, CASP-19 dependent:<br>Model 1:<br>Frailty B coefficient = -35.3 (-38.0 to -32.5);<br>$R^2$ = 0.362<br><br>Model 2:<br>Frailty B=-34.4 (-37.1 to -31.6);<br>$R^2$ = 0.376 | Both models: age, sex, smoking, and level of physical activity.<br>Model 2 also: net financial wealth and net income.<br>Models weighted to compensate for survey non-response and to take into account the survey's complex clustering and stratification. |
| Freitag 2016<br><br>N=210<br>Frailty: Tilburg<br>QoL: SF-12 and EUROHIS-8 | n=123<br><br>QoL scores not reported by frailty category | n=0[c] | n=87 | Correlation coefficents:<br>EUROHIS-8: -0.562 ***<br>SF-12 PCS: -0.589 ***<br>SF-12 MCS: -0.450 *** | None |
| Gobbens 2012<br><br>N=479 (in 2008)<br>N=336 (in 2009)<br>N=266 (in 2010)<br>Frailty: Tilburg<br>QoL: WHOQOL-BREF | Number of participants not reported by frailty category<br><br>QoL scores not reported by frailty category | | | Correlation coefficients with TFI in 2008 (baseline):<br>2008 (baseline) / 2010 (+2 years)<br>Physical -.72*** / -.68***<br>Psychological -.68*** / -.59***<br>Social relationships -.39*** / -.37***<br>Environmental -.54*** / -.45***<br><br>Sequential regression analyses, 2010 QOL subscales dependent, 2008 independent variables:<br>$R^2$ increase for addition of frailty domains to model; $R^2$ for overall model:<br>Physical .037***; .64***<br>Psychological .044***; .56***<br>Social relationships .046**; .42***<br>Environmental .018*; .56*** | baseline of the QOL variable, gender, age, marital status, education, income, lifestyle, life events, chronic diseases |
| Gobbens 2013<br><br>N=1,031<br>Frailty: Tilburg<br>QoL: WHOQOL-BREF | n=745 (72%)<br><br>WHOQOL-BREF, mean(SD):<br>Physical 16.68 (1.82)<br>Psychological 15.06 (1.73)<br>Social 15.08 (2.29)<br>Environment 16.23 (1.78) | n=0[c] | n=286 (28%)<br><br>13.61 (2.35)<br>13.32 (1.86)<br>13.11 (2.57)<br>14.58 (2.04) | | |

| Study details | Numbers of participants and QOL scores by frailty category [a]<br>Fit † | Pre-frail ‡ | Frail § | Reported analyses of associations between frailty and QOL | Covariates adjusted for |
|---|---|---|---|---|---|
| Jurschik 2012 | n=227 (43%) | n=246 (47%) | n=50 (10%) | | |
| N=640 | SF-36 [including pre-frail - n=473 (90%)], mean (SD) | | | Between group differences (Student's t-test), p-value: | In logistic regression: age, gender, marital status, alcohol use, comorbidity, cognitive status, basic disability, instrumental disability, depressive symptoms, vision problems, malnutrition, self-perceived health, polypharmacy, falls in the last year, social relations and SF-36 scales except bodily pain |
| Frailty: Fried | Physical function 68.40 (26.3) | | 24.29 (24.4) | *** | |
| QoL: SF-36 | Role physical 82.75 (32.5) | | 42.35 (46.3) | *** [g] | |
| | Bodily pain 40.94 (15.8) | | 42.04 (19.7) | - | |
| | General health 68.40 (26.3) | | 24.29 (24.4) | *** | |
| | Vitality 58.03 (17.6) | | 40.82 (18.5) | *** | |
| | Social functioning 87.45 (21.3) | | 59.64 (23.9) [f] | *** | |
| | Role emotional 87.72 (29.8) | | 54.42 (48.4) | *** | |
| | Mental health 73.86 (22.2) | | 54.64 (29.7) | *** | |
| | | | | Logistic regression: p>.05 for each SF-36 domain [h] | |
| Kanauchi 2008 | n=77 (76%) | n=0 [c] | n=24 (24%) | | |
| N=101 | WHOQOL-BREF, mean ± SE: | | | Between group differences (ANCOVA), p-value: | Adjusting for factors such as age, creatinine clearance and depressed mood |
| Frailty: Hebrew | Physical 3.65 ± 0.06 | | 3.07 ± 0.12 | *** | |
| Rehabilitation | Psychological 3.61 ± 0.06 | | 3.11 ± 0.12 | *** | |
| Center for Aged | Social 3.30 ± 0.07 | | 3.14 ± 0.14 | - | |
| Vulnerability | Enviroment 3.59 ± 0.04 | | 3.08 ± 0.12 | *** | |
| Index | | | | | |
| QoL: WHOQOL-BREF | | | | | |
| Lahousse 2014 | n=1,216 (43%) | n=1,454 (51%) | n=163 (6%) | logistic regression, QOL as dependent variable | age and sex |
| N=2,833 | EQ-VAS, median [IQR] | | | frail vs. non-frail and intermediate frail group combined: | |
| Frailty: Fried | 80 [15] | 80 [15] | 70 [20] | *** | |
| QoL: EuroQol Visual analogue scale (EQ-VAS) | | | | | |
| Lenardt 2014 | n=49 (24%) | n=115 (57%) | n=39 (19%) | Between group differences (Kruskall-Wallis H), p-value; post-hoc by-group significance: | none |
| N=203 | SF-36, mean (SD) | | | | |
| Frailty: Fried | Physical function 87.7 (16.9) | 73.2 (24.5) | 61.1 (27.9) | ***; †>‡>§ | |
| QoL: SF-36 | Role physical 96.4 (16.1) | 80.4 (36.2) | 71.1 (41.1) | ***; †>(‡,§) | |
| | Bodily pain 78.9 (24.6) | 62.5 (31.8) | 60.4 (30.7) | **; †>(‡,§) | |
| | General health 76.0 (22.5) | 73.0 (22.7) | 71.4 (17.0) | - | |
| | Vitality 85.8 (17.2) | 75.9 (22.4) | 75.0 (24.4) | *; †>(‡,§) | |
| | Social functioning 89.9 (24.6) | 88.6 (25.3) | 85.6 (25.6) | - | |
| | Role emotional 89.5 (30.8) | 87.9 (31.1) | 81.1 (36.5) | - | |
| | Mental health 85.3 (16.7) | 77.9 (23.2) | 76.4 (23.4) | - | |

| Study details | Numbers of participants and QOL scores by frailty category [a] | | | Reported analyses of associations between frailty and QOL | Covariates adjusted for |
|---|---|---|---|---|---|
| | Fit † | Pre-frail ‡ | Frail § | | |
| Lin 2011<br><br>N=903<br>Frailty: Fried<br>QoL: SF-36 | n=426 (46%)<br><br>SF-36, adjusted mean ± SE<br>Physical function 85.60 ±1.32<br>Role physical 93.95 ± 2.67<br>Bodily pain 83.49 ± 1.34<br>General health 66.96 ± 1.57<br>Vitality 77.69 ± 1.51<br>Social functioning 95.94 ±1.21<br>Role emotional 94.38 ± 2.21<br>Mental health 82.31 ± 1.33<br>PCS 50.48 ± 0.53<br>MCS 56.22 ± 0.62 | n=415 (44%)<br><br><br>80.62 ±1.25<br>83.26 ±2.52<br>81.23 ±1.26<br>60.55 ±1.48<br>72.13 ±1.42<br>91.57 ± 1.14<br>87.28 ± 2.08<br>79.69 ±1.25<br>48.01 ±0.50<br>54.47 ±0.59 | n=92 (10%)<br><br><br>62.74 ± 1.96<br>78.16 ± 3.96<br>74.29 ± 1.99<br>49.22 ±2.33<br>63.15 ± 2.24<br>80.17 ± 1.79<br>85.90 ±3.28<br>73.67 ± 1.97<br>42.56 ±0.79<br>52.64 ±0.92 | Between frailty categories difference (ANCOVA):<br>F value; Multiple post-hoc comparison<br>56.46**; †>‡>§<br>12.13***; †>(‡,§)<br>8.89***; †>‡>§<br>25.95***; †>‡>§<br>19.45***; †>‡>§<br>32.45***; †>‡>§<br>7.01***; †>(‡,§)<br>8.17***; †>‡>§<br>43.61***; †>‡>§<br>8.10***; †>(‡,§)<br><br>Multivariate logistic regression. Odds ratio of "successful aging" (SF-36 MCS AND PCS score in the highest tertile: PCS>=53.00; MCS>=59.28)<br>Pre-frail vs. Fit 0.45 (0.24 to 0.84)<br>Frail vs. Fit 0.14 (0.02 to 1.13) | Age (years), Gender, education, money use, You see relatives/friends when you want, Visual capacity, hearing capacity, regular exercise, hypertension, heart disease, Hyperuricemia, Arthritis, Cataract, fall history, pain, sleep disorder |

| Study details | Numbers of participants and QOL scores by frailty category [a] | | | Reported analyses of associations between frailty and QOL | Covariates adjusted for |
|---|---|---|---|---|---|
| | Fit † | Pre-frail ‡ | Frail § | | |
| Masel 2009 | n=264 (26%) | n=547 (54%) | n=200 (20%) | | |
| N=1,011 | SF-36, mean (SD) | | | Between frailty categories difference (ANOVA): | |
| Frailty: Fried | Physical function 64.3 (27.7) | 44.5 (30.5) | 23.3 (24.2) | * | |
| QoL: SF-36 | Role physical 80.6 (37.0) | 54.0 (47.1) | 31.4 (43.2) | * | |
| | Bodily pain 77.2 (24.8) | 64.1 (29.5) | 49.7 (31.0) | * | |
| | General health 68.3 (17.3) | 57.7 (20.4) | 43.5 (20.9) | * | |
| | Vitality 72.9 (18.4) | 60.6 (22.1) | 44.6 (22.6) | * | |
| | Social functioning 88.6 (20.7) | 71.6 (30.5) | 47.8 (33.8) | * | |
| | Role emotional 94.6 (20.9) | 78.1 (40.2) | 52.8 (48.3) | * | |
| | Mental health 84.8 (14.9) | 78.0 (19.6) | 66.2 (21.4) | * | |
| | PCS 44.1 (10.4) | 36.2 (11.9) | 29.1 (9.9) | * | |
| | MCS 58.4 (6.3) | 54.5 (10.7) | 46.9 (12.7) | * | |
| | | | | Multiple linear regressions, QOL subscales as dependent variables. | Multiple linear regression |
| | | | | pre-frail β; frail β | adjusted for age, sex, education, |
| | | | | Physical function: -0.24 *; -0.44 * | marital status, financial strain, |
| | | | | Role physical: -0.25 *; -0.38 * | arthritis, chronic illnesses, and |
| | | | | Bodily pain: -0.18 *; -0.26 * | BMI |
| | | | | General health: -0.20 *; -0.41 * | Logistic regression adjusted for |
| | | | | Vitality: -0.22 *; -0.45 * | age, sex, marital status, |
| | | | | Social functioning: -0.24 *; -0.49 * | financial strain, arthritis, chronic |
| | | | | Role emotional: -0.20 *; -0.38 * | illnesses, and BMI |
| | | | | Mental health: -0.16 *; -0.33 * | |
| | | | | PCS: -0.26 *; -0.42 * | |
| | | | | MCS: -0.18 *; -0.40 * | |
| | | | | Logistic regression, odds ratios for the effect of frailty status on scoring in the lowest quartile of the SF-36 summary scales: pre-frail; frail | |
| | | | | PCS: 4.03 (1.95, 8.35); 10.58 (4.90, 22.84) | |
| | | | | MCS: 3.86 (2.07, 7.19); 10.20 (5.19, 20.07) | |
| Pinto 2016 | Number of participants not reported by frailty category | | | Pearson's correlation -0.175, | |
| N=2,164 | QoL scores not reported by frailty category | | | ANOVA F 14.117, ***; | |
| Frailty: Fried | | | | | |
| QoL: Life satisfaction (unreferenced multi-item) | | | | Path analysis, Satisfaction < = Frailty: estimate = -0.186, standard error [j] = 0.081 * Standardized Direct Effects = -0.047, Standardized Indirect Effects = -0.025, ** | Age, education, number of diseases, depressive symptoms, cognitive status, self-rated health |

| Study details | Numbers of participants and QOL scores by frailty category [a] | | | Reported analyses of associations between frailty and QOL | Covariates adjusted for |
|---|---|---|---|---|---|
| | Fit † | Pre-frail ‡ | Frail § | | |
| Simone 2013<br><br>N=95<br>Frailty:<br>Groningen Frailty Indicator<br>QoL: Satisfaction with Life Scale | n=51 (54%)<br><br>Life Satisfaction, mean (SD)<br>27.94 (4.66) | n=0 [c] | n=44 (46%)<br><br><br>22.76 (6.30) | Between frailty category means:<br>ANOVA F = 20.46, **<br><br>Canonical correlation analysis:-<br>“Functional status” latent variable from frailty, social leisure engagement and solitary leisure engagement; “Subjective well being” latent variable from life satisfaction, positive affect and negative affect:<br>Frailty -> Functional status latent variable r=.844<br>Functional status latent variable <-> Subjective well being latent variable r=.515 | In canonical correlation analysis:<br>Functional status latent variable from frailty, social leisure engagement and solitary leisure engagement<br>Subjective well being latent variable from Life atisfaction, positive affect and negative affect |
| St John 2013<br><br>N=988<br>Frailty:<br>Cumulative Deficit Model<br>QoL: Life Satisfaction terrible-delightful scale | n=1287 (78%)<br><br>QoL scores not reported by frailty category | n=0 [c] | n=371 (22%) | Linear regression models for each domain of life satisfaction as dependent variable:<br>B coefficient of frailty at time point 1:<br>Unadjusted; Adjusted<br>Health −0.64 *; −0.63 *<br>Finances −0.15 *; −0.15 *<br>Family −0.28 *; −0.23 *<br>Friendships −0.35 *; −0.27 *<br>Housing −0.25 *; −0.20 *<br>Recreation −0.45 *; −0.40 *<br>Religion −0.19 *; −0.20 *<br>Self-esteem −0.22 *; −0.18 *<br>Transportation −0.31 *; −0.23 *<br>Overall −0.41 *; −0.37 *<br><br>Life satisfaction at time point 2 (5 years later) as dependent variable:<br>B coefficient of frailty at time point 1:<br>Unadjusted; Adjusted<br>Health −0.77*; −0.70*<br>Finances −0.25*; −0.21*<br>Family −0.24*; −0.19*<br>Friendships −0.51*; −0.43*<br>Housing −0.14; −0.05<br>Recreation −0.48*; −0.42*<br>Religion −0.15*; −0.18*<br>Self-esteem −0.18*; −0.04<br>Transportation −0.53*; −0.44*<br>Overall −0.32*; −0.27* | Adjusted models adjusted for age, gender, education, and marital status |
| Wu 2013<br><br>N=699<br>Frailty: Other<br>QoL: CASP-19 | Fit: n=80 (11%); Well: n=165 (24%)<br><br>CASP-19, mean (SD)<br>Overall Fit:41.7 (7.0); Well:40.5 (5.8)<br>- Control Fit: 7.4 (1.6); Well: 7.2 (1.3)<br>- Autonomy Fit:11.8 (1.5); Well:11.3 (1.5)<br>- Self-realisation Fit:12.4 (2.9); Well:12.5 (2.5)<br>- Pleasure Fit:10.2 (3.1); Well: 9.5 (2.7) | Vulnerable: n=437 (63%)<br><br><br>36.8 (7.0)<br>6.4 (1.4)<br>10.3 (2.0)<br>11.5 (2.9)<br>8.5 (2.6) | Frail: n=17 (2%)<br><br><br>34.1 (8.9)<br>7.1 (2.6)<br>9.1 (2.8)<br>10.5 (3.4)<br>7.4 (2.5) | <br><br>Between frailty category differences (ANOVA):<br>**<br>**<br>**<br>**<br>** | Not reported |

| Study details | Numbers of participants and QOL scores by frailty category [a] | | | Reported analyses of associations between frailty and QOL | Covariates adjusted for |
|---|---|---|---|---|---|
| | Fit † | Pre-frail ‡ | Frail § | | |
| Yang 2016<br><br>N=1,970<br>Frailty: Cumulative Deficit Model<br>QoL: Life satisfaction (unreferenced multi-item) | Number of participants not reported by frailty category<br><br>QoL scores not reported by frailty category | | | Linear regression, life satisfaction as dependent variable:<br>frailty index: B -23.85*** 95% CI(-26.67 to -21.03), SE 1.44, β -0.46***.<br>Frailty index x Old-old: B = 5.17* 95% CI(1.18 to 9.16), SE = 2.04, β = 0.07*<br>$R^2$ = 35.8% | urban/rural; gender; social vulnerability; old-old vs. Young-old; frailty x old-old |

[a] Participants per frailty group are presented as n= number (percentage of whole sample). QOL scores are presented as one of mean (standard deviation [SD]), mean ± standard error (SE), mean {95% confidence interval (95%CI)} or median [interquartile range (IQR)] for continuous variables and n= number (percentage of frailty group) for categorical variables.
[b] Only people living with frailty were included.
[c] The study only reported data in two frailty categories: Not frail and Frail. Data for Not frail participants is reported in the Fit (or robust) column.
[d] The Pleasure scale of the CASP-19
[e] The Control, Autonomy and Self-realisation scales of the CASP-19
[f] In Jurschik 2012, SF-36 social functioning for frail participants is reported as 59.64 (3.9), which is an infeasibly small standard deviation and could not be correct given the overall sample standard deviation. We have assumed this is 23.9.
[g] In Jurschik 2012, between groups p-value for SF-36 Role physical is reported in the table as 0.58 but this is contradicted in the text "the HRQOL indices (p < 0.001), with the exception of BP [bodily pain], were higher in frail than in non-frail participants" and does not fit with the means and standard deviations reported. We have corrected this to p < 0.001.
[h] In Jurschik 2012, the methods imply that each SF-36 domain except bodily pain was entered into a logistic regression model (bodily pain not significantly different between groups). The only results reported from the logistic regression are for statistically significant variables. Therefore, we assumed that p>.05 for each SF-36 domain except bodily pain in the logistic regression model, although this is not explicitly reported.
[j] In Pinto 2016, "SE" column heading is explained as "standardized estimates", but the values reported equate with standard errors when compared to the estimates and p-values, and standardised estimates are reported elsewhere in the table. Therefore, we have corrected this to standard error.
† Fit (or robust)
‡ Pre-frail (or vulnerable)
§ Frail (or moderate-severe frailty)
[-] p>0.05
[*] p≤0.05 Note that studies may not have reported specific p-values or the ranges adopted here. Therefore an indication of p≤0.05 may not preclude p≤0.01 or p≤0.001 for example.
[**] p≤0.01
[***] p≤0.001
